# Phanerozoic icehouse climates as the result of multiple solid-Earth cooling mechanisms

**DOI:** 10.1126/sciadv.adm9798

**Published:** 2025-02-14

**Authors:** Andrew S. Merdith, Thomas M. Gernon, Pierre Maffre, Yannick Donnadieu, Yves Goddéris, Jack Longman, R. Dietmar Müller, Benjamin J. W. Mills

**Affiliations:** ^1^ School of Physics, Chemistry and Earth Sciences, University of Adelaide, Adelaide, SA, Australia.; ^2^ School of Earth and Environment, University of Leeds, Leeds LS3 9JT, UK.; ^3^ School of Ocean and Earth Sciences, University of Southampton, Southampton, UK.; ^4^ Aix-Marseille Univ, CNRS, IRD, INRA, Coll. France, CEREGE, Aix-en-Provence, France.; ^5^ Géosciences-Environnement Toulouse, CNRS-Université Paul Sabatier, Toulouse, France.; ^6^ School of Geography and Environmental Sciences, Northumbria University, Newcastle-upon-Tyne, UK.; ^7^ EarthByte Group, School of Geosciences, University of Sydney, Sydney, NSW, Australia.

## Abstract

The Phanerozoic climate has been interrupted by two long “icehouse” intervals, including the current icehouse of the last ~34 million years. While these cool intervals correspond to lower atmospheric CO_2_, it is unclear why CO_2_ levels fell, with hypotheses suggesting changes in CO_2_ degassing rates or modification of silicate weathering through changing continental lithology or paleogeography. Here, we construct an Earth System Model that integrates these proposed cooling mechanisms in detail. The model can reproduce the broad geologic record of ice cap expansion, allowing us to infer the primary drivers of long-term climate change. Our results indicate that recent icehouse climates required a combination of different cooling mechanisms acting simultaneously and were not driven by a single known process, potentially explaining why icehouses have been rarer than greenhouses over Earth history.

## Teaser

Earth’s climate swings between greenhouse and icehouse states, with icehouses occurring when multiple cooling mechanisms combine.

## INTRODUCTION

### Long-term drivers of climate

Atmospheric CO_2_ levels control global temperature variations over geologic time, with the concentration of CO_2_ over these timescales determined by the long-term carbon cycle (*1*–*3*). Several fundamental tectonic drivers of the carbon cycle over 10– to 100–million year (Ma) timescales have been proposed over the past 40 years, which tend to be partly dependent on one another. Degassing of Earth’s lithosphere adds CO_2_ to the atmosphere (*4*–*6*). Weathering of silicate minerals—especially those exposed in continental arcs (*5*, *7*, *8*) or ophiolite-bearing suture zones (*9*)—removes CO_2_ from the atmosphere and ultimately buries it as carbonate minerals (*10*). Weathering also liberates nutrients needed for photosynthesis, which draws down atmospheric CO_2_ and sequesters carbon as organic matter (*11*–*13*).

Changes to Earth system through the Phanerozoic have fundamentally modified how these burial, degassing, and weathering processes operate. The most notable example is the evolution and colonization of Earth’s surface by land plants during the Silurian-Devonian, which irrevocably changed both the carbon cycle and weathering processes (*14*–*16*). Since this enhancement of weathering intensity and organic carbon burial by the emergence of land plants, subsequent variations in the carbon cycle are thought to have been driven primarily by the paleogeographic and paleotectonic evolution of Earth’s continents. The slow movement and shifting of the continents have caused variations in the CO_2_ degassing flux (*17*), changed patterns of global precipitation and runoff (*18*), and exposed different lithologies to enhanced weathering in the tropics (*7*–*9*, *19*, *20*). The intersection of these different mechanisms is hypothesized to control the overall degassing and weathering fluxes that set surface temperatures on Earth. Many studies have sought to reconstruct these processes over the Phanerozoic, and a succession of “Earth System” or biogeochemical models has attempted to integrate some combination of these mechanisms to reproduce the Phanerozoic CO_2_ or temperature record and identify the drivers of key climatic excursions (fig. S1) (*2*, *21*–*24*).

Empirical studies of the long-term carbon cycle have linked the temporal occurrence of continental arc degassing to climate warmth (*5*), their weathering to cooling (*8*), and the occurrence of low-latitude ophiolite bearing sutures have also been linked to cooling events (*9*). Modeling studies have suggested that mountain uplift events could also have driven climatic cooling (*25*, *26*). However, there remains little agreement on which processes are most important for causing icehouse intervals as we lack a continuous Earth System Model (ESM) that can disentangle how all these processes combine to drive observed changes. Continuous, global biogeochemical models for the Phanerozoic, such as GEOCARB(SULF) (*2*, *22*) and COPSE (Carbon-Oxygen-Phospherous-Sulfur Evolution) (*21*, *27*) have attempted to integrate these weathering and degassing processes but are nondimensional and not spatially explicit and therefore cannot track the spatial distribution of weatherable lithologies or hydrology through time. Furthermore, they are unable to spatially resolve global temperature. As a result of these limitations, previous models cannot adequately reproduce the cycles between icehouses and greenhouses evident in the geologic record (fig. S1). Here, we use plate tectonic modeling and global lithological maps to reconstruct the spatial and temporal variation of solid-Earth degassing rates and weatherable lithologies from subduction-related environments. We then incorporate these variables into a long-term 3D ESM [pySCION—a version of the SCION (Spatial, Continuous Integration) model (*23*) written for the python programming language] to assess the relative importance of the cooling mechanisms they represent. Because of the considerable uncertainty in the biogeography and biotic weathering ability of early plants, alongside the unusually short duration and substantial extent of the Hirnantian glaciation (*28*), we do not currently expand this analysis into pre-Devonian times. Although a dynamic terrestrial and marine biosphere and their organic carbon burial fluxes are included in our analysis, we do not assess here how plant colonization affected Earth.

## RESULTS AND DISCUSSION

### Assessing carbon sources and sinks over geologic time

Our ESM integrates a detailed reconstruction of the global CO_2_ degassing rate with the global paleogeography and distribution of weatherable lithologies on the continents, overcoming the limitations of previous approaches. Degassing from the Earth’s mantle and lithosphere is considered the main source of atmospheric CO_2_ over million-year timescales (*6*). Degassing estimates have traditionally been taken from a variety of proxies (*1*), but recently full-plate reconstructions made using GPlates have allowed for quantitative four-dimensional (4D) approach through the mapping of dynamic processes at subduction zones over Phanerozoic time. Using this framework, a recent study coupled thermodynamic modeling to subducted carbon inventories to quantitatively assess the mobility of carbon in downgoing slabs and degassing through the Mesozoic and Cenozoic (*6*). Crucially, this study was able to quantify the contribution of pelagic sediments to arc-degassing over time, which increased markedly with the evolution of pelagic calcareous nannoplankton during the Cretaceous Period. Thus, our implementation of it as part of our degassing curve allows us to remove from our model the “burial depth factor,” an arbitrary scaling of degassing for pre-Cenozoic times used in all carbon-cycle models (*29*) that represents the increasing contribution to degassing budgets from pelagic calcareous nanoplankton since the Cretaceous.

**Fig. 1.**
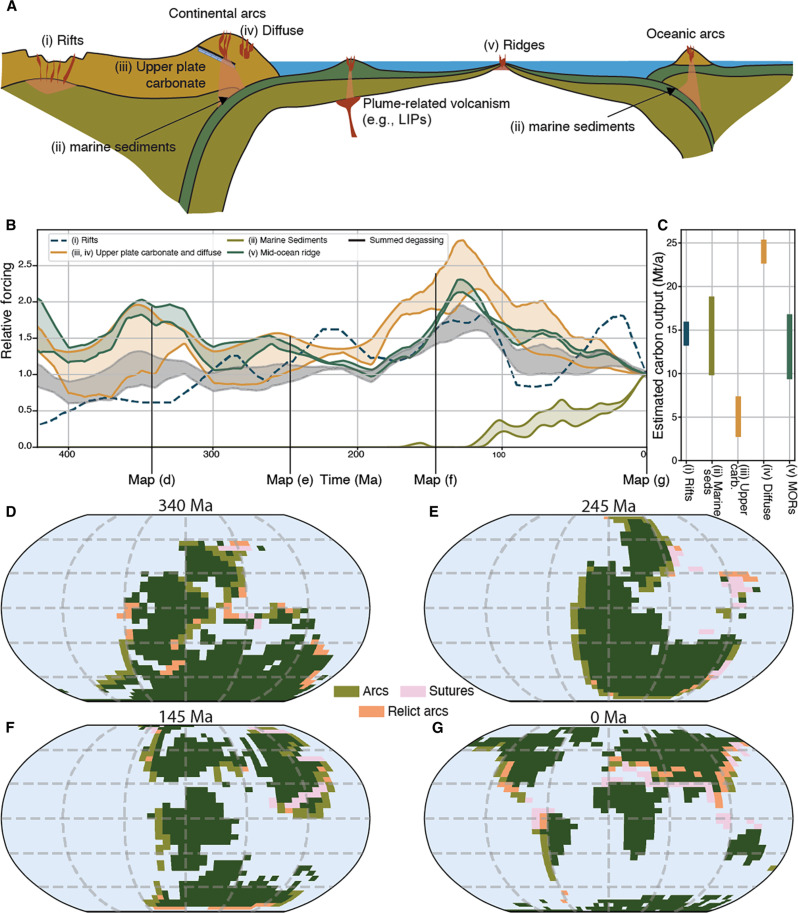
Sources and sinks of CO_2_ implemented within pySCION. (**A**) Summary of different tectonic environments from which carbon is degassed. (**B**) Tectonic forcing curves that we use to scale each individual flux back in time divided into continental rifts, continental arcs, mid-ocean ridges, and subducting marine sediments. Our total summed curve used to drive the model is also depicted. (**C**) Present-day measured or modeled flux from different sources [corresponding to (A), including from (i) continental rifts (*97*, *104*); (ii) from subduction of marine sediments (*6*, *110*); (iii) assimilated from lower crustal sources on the upper plate (*106*); (iv) diffuse degassing from the flanks of continental arcs (*104*); and (v) from mid-ocean ridges (*6*)]. (**D** to **G**) Spatial boundary conditions (land-sea/paleogeographic maps) (*119*, *120*) of pySCION, depicting our lithological maps that are used to enhance the silicate weathering process.

Our analysis spans the Phanerozoic and includes degassing estimates from mid-ocean ridges, continental arcs, and continental rifts (Fig. 1A). We compile estimates of present-day fluxes from the literature (see Materials and Methods, data S1, and Fig. 1, B and C) and scale them to relevant tectonic processes to estimate how the carbon flux in different tectonic environments have changed through time (Fig. 1B). We use estimates of seafloor production and consumption taken from full-plate reconstructions to scale contributions from mid-ocean ridges and subduction zones and use the lengths of pericontinental subduction zones extracted from a full-plate model (*30*) to scale the amount of carbon that could be released at continental-arc environments.

**Fig. 2.**
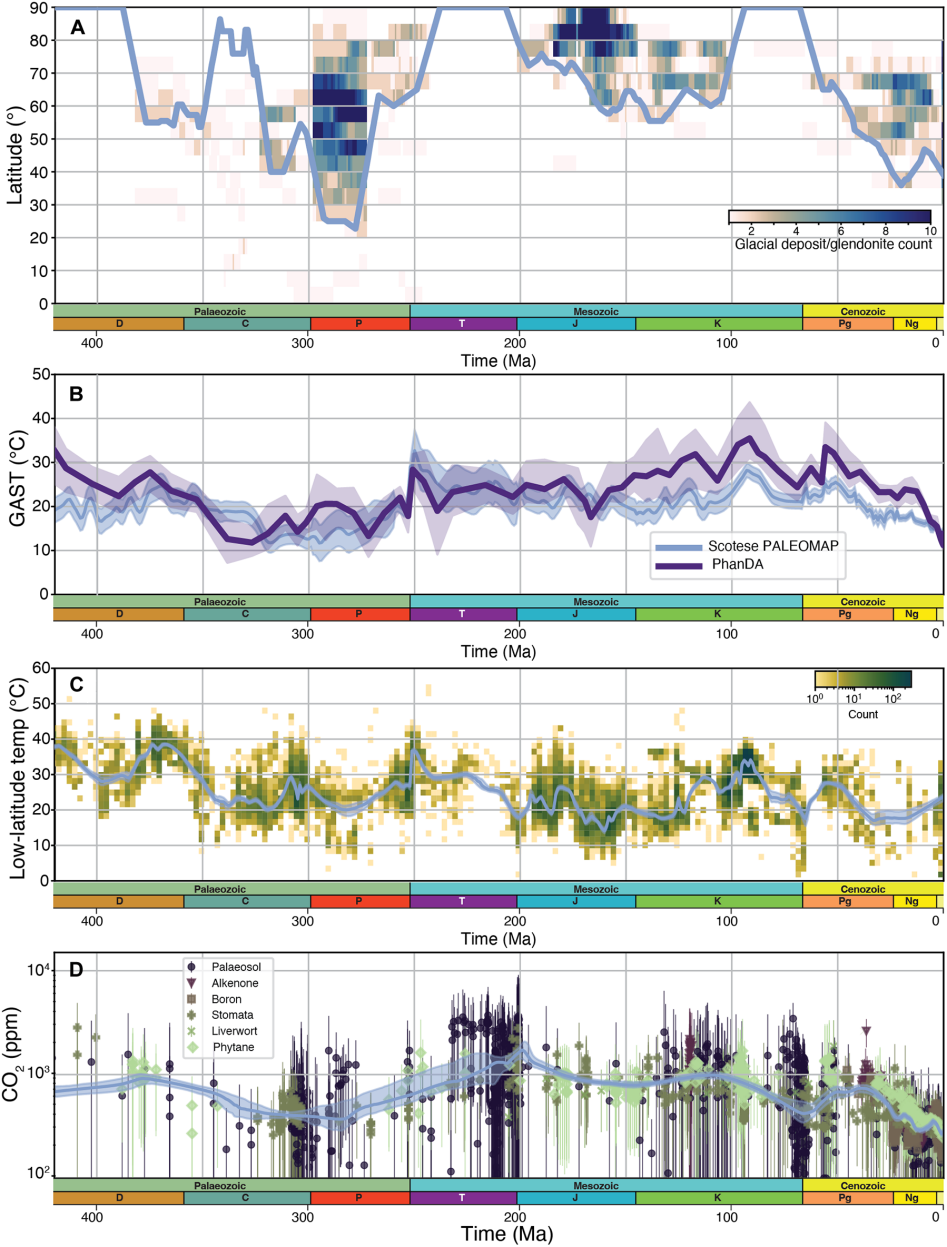
Overview of proxy data used to constrain paleosurface conditions. Lines of best fit are shown in each panel (in blue). (**A**) Ice line extent estimated from preserved lithological indicators of cold climates (tillites, dropstones, and glendonites). The heatmap shows the temporal distribution in 10° latitudinal bins, considering the age uncertainty provided in the underlying dataset, and is colored by abundance. The maximum ice extent is a smoothed line (moving window of 11 Ma) requiring at least two or more deposits to be in a latitudinal bracket (explaining why in the Carboniferous this does not extend to 90°). For (C) and (D), a line of best fit and 95% confidence interval (solid blue line and interval) was calculated using a nonparametric locally weighted scatterplot smoothing (LOWESS) function (www.statsmodels.org). (**B**) GAST with uncertainty (blue line and envelope) in (*45*, *46*) and GAST with uncertainty (2 sigma, indigo line and envelope) in (*47*). (**C**) Equatorial average surface temperature derived from oxygen isotopes (*51*). We have depicted the data as a heatmap to show the concentration. (**D**) pCO_2_ proxy data (*57*, *58*). Ma, million years.

We deliberately omit degassing estimates from large igneous provinces (LIPs), including siliceous LIPs (sLIPs) from our compiled degassing curve, as their individual volcanic activity (and inferred degassing contribution) typically spans only a few million years (*31*, *32*). In contrast, our focus is the 10– to 100–milion year (Ma) variability recorded in proxy records. This does not discount the contribution to warming or perturbations to the Earth system by either LIPs or sLIPs on short (<5 Ma) timescales, where they likely cause substantial change (*33*, *34*). Example model runs using a basic LIP degassing database (*35*) are shown in the fig. S14, which underscore this point. They also demonstrate how longer-lived LIPs should have lower average degassing rates, so while some may degas over intervals of >10 Ma, the modeled climate on these timescales does not show appreciable long-term changes. The degassing contribution of the Siberian Traps, which is the only LIP we include in our “default” model runs as it supplied an order of magnitude more CO_2_ than any other Phanerozoic LIP (*36*–*38*), still only led to elevated surface temperatures for <5 Ma, which are shown in the temperature proxy record (Fig. 2, B and C).

Figure 1B depicts our total carbon degassing flux (shaded blue area, black outline) relative to present-day levels. It shows (i) high CO_2_ degassing during the Cretaceous; (ii) modern levels during the late Paleozoic and Early Mesozoic, and (iii) below present-day levels in the Devonian. Our uncertainty range is calculated by dividing the minimum and maximum limits from the composite curve by the 10th and 90th percentiles (respectively) at present-day (see Materials and Methods for details). In our merged curve, diffuse degassing around continental arcs and degassing from ridges and rifts are the most significant contributors to CO_2_ over our analysis (Fig. 1 and fig. S2), although at present-day their contributions broadly overlap with degassing derived from subducted pelagic sediments (Fig. 1C). Tracking these data back through time underscores the importance of quantifying continental deformation (that is, mainly arcs and rifts) through time, as these regions plausibly contribute between 40 and 60% of the total solid-Earth degassing budget at any time (Fig. 1, B and C).

In the original SCION model (and in GEOCARBSULF and COPSE), Earth’s surface lithology is globally uniform, and a fraction of the continental area is assumed to be volcanic (*35*, *39*). For the current model, we incorporate a digital database of ophiolite-bearing sutures (*9*) and estimates of peri-continental subduction zones (inferred to represent the possible extent of continental arcs) extracted from a plate model (*30*). These are two distinct but overlapping lithotectonic features, with continental arcs being the surface expression of subduction-driven volcanism and ophiolitic-bearing sutures being obducted oceanic crust when two pieces of continental crust collide. For each time step, we produce a grid overlaying the paleogeography that records the fractional content of each cell that contains either arcs (primarily intermediate rocks) or ophiolite-bearing sutures (primarily ultramafic-mafic rocks) (see the Materials and Methods; Fig. 1, D to G, and fig. S4). To include important but tectonically inactive lithological and topographic features, we propagate each raster grid of active pericontinental subduction zones by one grid time step (~20 Ma), thus capturing the effects of “relict arcs” (*7*). Once the arc, suture, and relict-arc grids were constructed, the contributions of each to silicate weathering through time were calibrated using an arc and suture “enhancement factor” (AEF and SEF, respectively), a multiplier applied to the fractional content of the respective grids (the AEF is also applied to the relict arc grids). Weathering enhancement factors were calculated using a least-squares optimization on the weathering equations (*40*) at present-day and determining the enhancement factors necessary to best match the riverine silicate flux across major world rivers (*41*). This analysis found an AEF of 7 and SEF of 20 best reproduced the present-day data (table S1 and fig. S4).

### Phanerozoic proxy data

To evaluate our model, we consider four different types of proxy data (Fig. 2) that can be used to infer transitions between icehouse and greenhouse conditions: (i) ice cap latitude, (ii) global average surface temperature (GAST), (iii) low-latitude sea surface temperature, and (iv) *p*CO_2_ levels. We consider ice cap extent to be the most robust indicator of icehouse-greenhouse transitions, but because no proxy data are able to provide a perfect indication of global temperature, we present our results against all four datasets. For each of these datasets, we add a line of best fit to compare our model quantitatively, although because of data coverage, some datasets are more amenable to this than others.

Our ice cap latitude estimates combine a database of glacial deposits (*42*) and Phanerozoic glendonites (*43*), reconstructed to their paleocoordinates using a full-plate model (*30*). Uncertainties in this dataset include the reconstructed latitudes (*44*) and the potential that glendonite formation might reflect the upwelling of cold water rather than cold surface temperatures (*43*). Figure S4 depicts both datasets separately rather than combined, as in Fig. 2A.

GAST estimates are taken from two recent compilations: first, from Scotese *et al.* (*45*), with uncertainty from Van der Meer *et al.* (*46*), who use maps of climate-sensitive lithologies through time to define paleo-Köppen belts, with shorter-term variations based on oxygen isotopes. The continuous nature of this record, and its normalization against long-term changes in seawater oxygen isotope ratios, makes it an attractive benchmark against which to compare model results. In addition, uncertainty in reconstructed latitudes, this method relies on the assumption that Köppen belts, and their indicators are broadly consistent over time (Fig. 2B). We also compare our results to the most recent “PhanDA” curve (*47*), which uses a data assimilation approach to combine temperature estimates from δ^18^O and palaeoclimate models to estimate GAST. Although this record does not correct for variations in seawater δ^18^O (*48*), there is reasonable consensus between both approaches with regard to the timings and durations of icehouses and greenhouses despite some differences in global average temperature. The most notable deviations pertain to the trajectory of late Palaeozoic cooling between the late Devonian and late Carboniferous and the magnitude of warmth during the Cretaceous and early Cenozoic, where estimates from (*47*) are ~5° to 10°C warmer than those of (*45*) (Fig. 2B). We also plot the low-latitude ±30° sea surface temperature proxy from δ^18^O of shelly marine fossils (*49*, *50*). The data we use here predominantly come from a recent compilation (*51*), supplemented by studies focusing on more discrete time periods (Fig. 2C) (*52*–*55*). As with PhanDA, a key uncertainty with this proxy is whether predicted warm ocean temperatures in the Early Paleozoic are realistic or whether they reflect changing oxygen isotope composition of seawater (*3*, *50*, *56*).

A range of proxies are used to constrain past CO_2_ levels based on either photosynthesizing organisms (e.g., stomatal density in leaves, δ^13^C in liverworts, and alkenones) or on seawater pH through boron isotopes in foraminifera and pedogenic carbonates. We use three recent compilations (*57*–*59*) that provide proxy data back to the Ordovician. Uncertainty ranges for these proxies are based on different methodological steps, particularly the estimation of soil CO_2_ levels for the paleosol record (*60*) and the boron isotopic composition of seawater for reconstructing paleo-pH (*61*).

### Modeled Phanerozoic climate and driving mechanisms

**Fig. 3.**
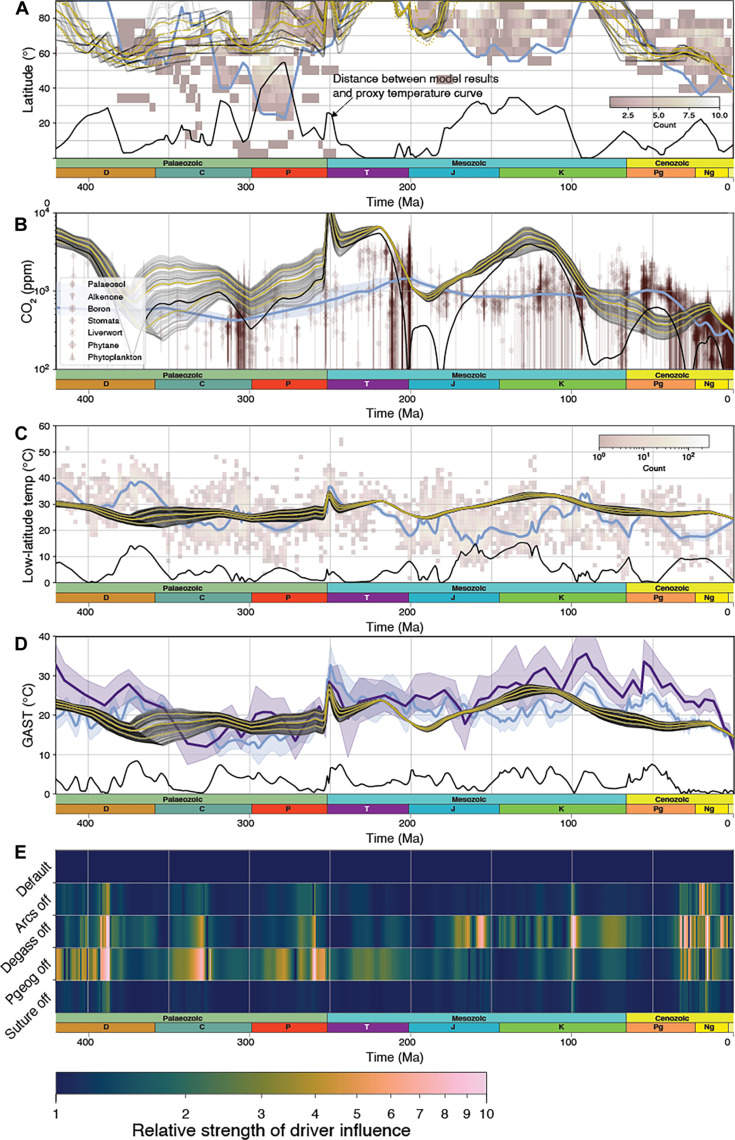
pySCION model results for the Phanerozoic. Results from our default run are plotted in (A) to (D), where gray lines show 10% of the ensemble (1000 runs per ensemble), and the solid amber lines are the mean ± 1 SD. In some cases where ensemble runs overlap, the lines are black [e.g., (A)]. For comparison, proxy data are in red-brown, and their lines of best fit and confidence are shown in blue (see Fig. 2 caption for details). Solid black lines show the distance between proxies and model. (**A**) Ice line results. The default model run assumes a critical temperature of −10 °C, and the mean values of alternative runs assume critical temperatures of −8° and −12°C that are represented by the coarse and fine dashed amber lines, respectively. (**B**) Atmospheric CO_2_ results. (**C**) Modeled equatorial (±30°) average surface temperature against equatorial (±30°) sea surface temperature from δ^18^O (*51*). (**D**) GAST and uncertainty of Scotese *et al.* (*45*) in blue and Judd *et al.* (*47*) in indigo. (**E**) Relative influence of each climate driver when it is turned “off” (no change relative to present-day)—a measure of how influential each driver is through time. Temperature, ice line, and CO_2_ for each series of runs are provided in figs. S9 to S14. Ma, million years.

We now run the SCION model through the Phanerozoic. CO_2_ degassing follows our compiled curve in Fig. 1B, and weatherable lithologies are mapped onto the model land surface (Fig. 1, D to G). We evaluate our model by comparing its prediction of the geographic extent of ice sheets against the proxies from Fig. 2A, with a secondary focus on surface temperatures and CO_2_ levels in Fig. 1 (B to D), which are less certain. SCION assumes that an ice sheet is present if the grid cell continental temperature of the model output has a mean annual temperature below −10°C (e.g., *62*); as a sensitivity test, we also plot the predicted ice curve for −12°C and −8°C. Figure 3 shows our “default” model ensemble, which includes all processes and considers the uncertainty bounds in the CO_2_ degassing rate and uncertainty in the background weatherability of the non-arc and nonsuture continental grid cells through time. We also show the calculated Wasserstein Distance—the mean distance between two distributions—between the uncertainty range of our results and that of the proxies in each panel.

We find that the model (Fig. 3) can replicate several key features of the Phanerozoic ice cap paleolatitude record (*42*), showing cooling from a warm early Devonian to a Carboniferous-Permian icehouse, followed by warming into a Triassic greenhouse, cooling through the Jurassic cool interval, into a Cretaceous hothouse and lastly into a Cenozoic icehouse (*63*–*65*). The model produces more mixed fits to the CO_2_ proxy record, compiled global average temperature record (*45*, *47*), and low-latitude oxygen isotope record, although the uncertainty in these records is much higher. Key misfits in the combined records, identified by the model-proxy distance metrics shown at the bottom of each panel, occur during (i) the Devonian—where ice cap expansion and CO_2_ drop are roughly matched, but temperature proxies are not (max ~10 °C distance); (ii) the Permian, where temperature proxies are well-matched, CO_2_ proxies are highly variable, but the model ice cap extent is located at higher latitudes than the associated proxy record (max ~50° distance); (iii) the Early Cretaceous, where the model has temperature within the wide uncertainty in different reconstructions but has higher CO_2_ [and max ~3000 parts per million (ppm) CO_2_ distance] and has more restricted ice caps than the geologic record suggests; and (iv) the Eocene, where the model is colder and has lower CO_2_ than the proxy records suggest. The estimation of ice sheet extent in our model is likely complicated by the underlying paleogeographic maps. For example, the mismatch in ice expanse during the Permian is possibly because the underlying paleogeographic-topographic model is not conducive to producing low temperatures at low latitudes, where, at this time, localized and regional topography may be more important. We note that our model predicts the iceline extending to ~60° latitude, which is reasonably consistent with the bulk of the Gondwanan ice sheet (*66*). Our results over the Early Triassic also differ from the GEOCLIM model (*67*) because we add a weathering dependency on erosion and also have abundant low- to mid-latitude ophiolitic sutures at this time that appear to help drive some component of Early Triassic cooling (fig. S6).

Overall, despite some apparent misfits, our model makes a generally reasonable prediction of long-term greenhouse-icehouse trends at the 10- to 100-Ma scale within the uncertainty offered by multiple different proxy datasets. It performs better [with a mean Wasserstein distance to modeled GAST (*45*) of ~3.3] than the original SCION model (Wasserstein distance of 4.07) and previous simpler and more idealized model frameworks (e.g., GEOCARBSULF and COPSE, Wasserstein distances of 3.41 and 4.57 respectively; fig. S1). We now seek to better understand what is driving these model predictions by examining how individual processes act to warm or cool the model climate. To do this, we systematically rerun the model with individual drivers turned off to quantify the influence of each individual driver on changing the modeled GAST curve relative to the default GAST curve of (Fig. 3D) (*45*)—we choose this proxy curve as a reference for this section because (i) it is continuous in high resolution over the Phanerozoic, (ii) it does not reach a maximum beyond which it can no longer vary (e.g., 90° for the ice line), and (iii) it is robust to changing seawater oxygen isotope compositions. We assess the impact of the climate drivers by calculating the ratio between the Wasserstein Distance of the default model distribution and each individual run with a driver switched off, with the temperature distribution at each time step. This allows us to test the influence of several previously suggested drivers of Phanerozoic Icehouses (Fig. 3E) (*5*, *14*, *25*).

In our model, the Devonian-Carboniferous cooling is predominantly driven by changes in paleogeography and a decrease in degassing, although arc weathering also plays a prominent role (Fig. 3E). Both changes in palaeogeography and degassing have been individually proposed to initiate glaciation (*5*, *14*, *25*). The former through a reduction in sea level (*68*, *69*) and emergence of continental shelves and islands (*70*–*72*) and the latter by a reduction in degassing that has been linked to a slowdown in subduction rates as inferred by a lull in the magmatic zircon record (*5*) triggering cooling. However, our model suggests that it is only through a combination of these processes acting together that an extensive icehouse could be initiated (figs. S5 to S8). Our results suggest that once initiated, the prolonged icehouse was maintained predominantly by the paleogeographic configuration of Earth, wherein the low-latitude Variscan-Hercynian Orogeny (which formed in the Permian from the collisions between Gondwana and Laurussia to form Pangea) increased physical weathering, maintaining cool temperatures (*25*).

The warm Mesozoic climate state in our model is also most sensitive to changes in degassing and paleogeography, specifically the increase in volcanic degassing driven by the initial rifting stages of Pangea breakup between 245 and 200 Ma. Here, we recognize an interplay between both mechanisms, whereby degassing of continental rifts increases the build-up of *p*CO_2_. However, this degassing occurs without forming new ocean basins, thus, in a palaeogeographic sense, preventing an increase in supply of nutrients derived from chemical weathering (fig. S6) reaching young ocean basins, resulting in warming (although our model is not yet able to capture the full extent of this as weathering products are always assumed to reach the ocean). Arc systems and ophiolitic sutures still contribute to enhanced silicate weathering (*8*); however, their contribution does not change substantially over the Mesozoic as their spatial footprint remains mostly constant, and their exposure is often balanced by hydrological changes (i.e., less rainfall and runoff). Our modeled warmer Mesozoic climate is punctuated by ephemeral glaciation in the Jurassic, in reasonable, but not perfect, agreement with the geologic record of glacial deposits at this time (*42*). Our analysis suggests that this short-term decrease in global temperature (evident in all three temperature proxy curves) and increase in high-latitude glacial deposits is mostly driven by a reduction in degassing from both rifts and mid-ocean ridges (Fig. 1) and an increase in erosion and weathering following the breakup of Pangea (figs. S6 and S7).

Last, cooling during the Late Cretaceous and Cenozoic is most sensitive to our modeled decrease in solid-Earth degassing (Fig. 2), alongside changes to paleogeography and arc weathering. In our compiled degassing curve, the decrease is principally driven by the reduction in degassing around continental arcs, as the decrease in mid-ocean ridge degassing is offset by the increase in degassing driven by the subduction of pelagic sediments over this time (*6*). Tighter quantifications of the contributions of diffuse degassing around arcs and rifts would potentially change these sensitivities. Changes in paleogeography, such as those resulting from the closure of the low-latitude Tethyan Ocean and formation of the Himalayas as well as exposure of arcs and ophiolitic sutures around Southeast Asia, also played a role in increasing the efficacy of the silicate weathering cycle (fig. S8). While previous studies have proposed each of these drivers acting in isolation to explain this climate shift (*5*, *19*, *26*), our model results suggest that all three played important roles in contributing to global cooling.

### No single driver for Phanerozoic icehouses

**Fig. 4.**
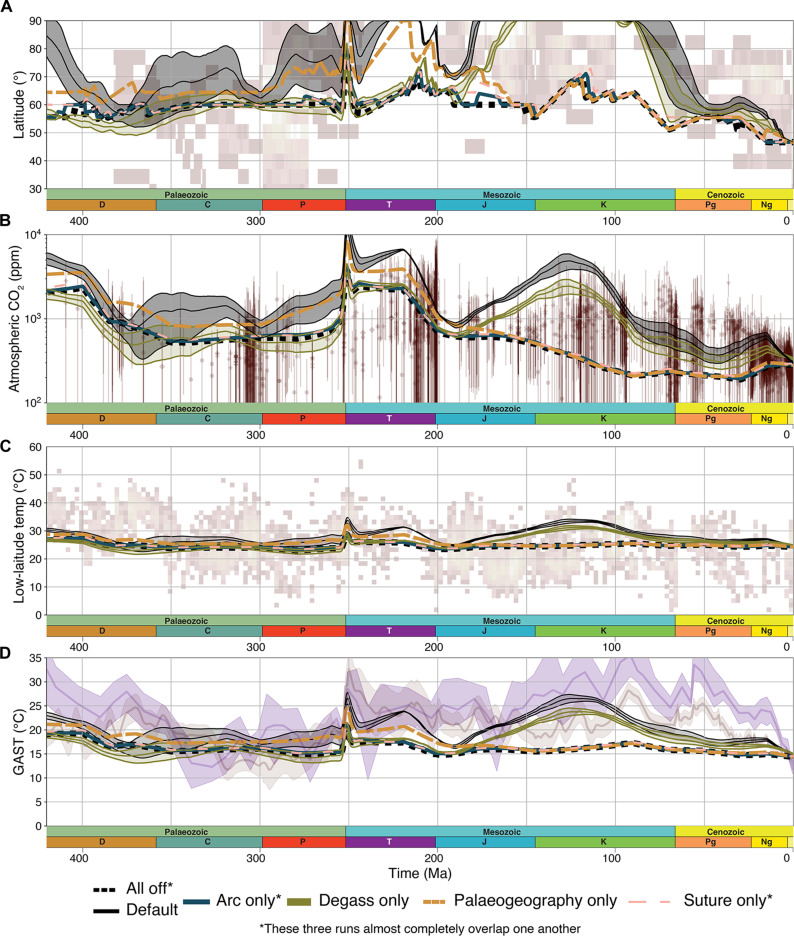
Results of the model where only one driver is turned on. Proxy data in each panel is the same as those shown in Fig. 3. The default run (all drivers on) is plotted as black lines with gray-shaded area. The figure shows (**A**) ice line, (**B**) pCO_2_, (**C**) equatorial average surface temperature, and (**D**) GAST of Scotese *et al.* (pale red) (*45*) and Judd *et al.* (*47*) (indigo). “Arc” and “suture” only refer to the modeled contribution to silicate weathering of continental arcs and ophiolitic bearing sutures, respectively. Note that organic carbon burial is still active in these runs. Ma, million years.

To further explore whether any of the key proposed processes could exert a first-order control on Phanerozoic climate, we rerun the model subject to a single climate driver (Fig. 4 and figs. S9 to S13). As a baseline reference, we ran the model with all drivers turned off. As expected, this produces a predominantly icehouse Earth similar to that of the late Cenozoic (black dashed lines in Fig. 3 and fig. S7). We find that when we run the model systematically with only each discrete driver, the model produces an overall poor fit for the Phanerozoic icehouse-greenhouse record. While the weathering of volcanic arcs is important, it does not change substantially over the Phanerozoic and, therefore, is unable to independently force major climate change (Fig. 4 and fig. S15). Weathering of ophiolite-bearing sutures changes substantially but constitutes only a minor component of the total weathering budget (Fig. 4 and fig. S15).

Degassing and paleogeography show a greater ability to drive climatic change when considered alone. While some of these model runs yield reasonable fits for certain time periods, we observe numerous discrepancies with the climate record when each driver is considered in isolation (Fig. 4). Notably, these discrepancies are far greater than those observed in the “combined drivers” model (Fig. 3). In particular, the “degassing only” run can reproduce a Cretaceous hothouse, but it is unable to reproduce a warmer Triassic (Fig. 4). No single mechanism can reproduce a warm Devonian, with all four single-driver runs producing an ice-dominated Devonian world (Fig. 4A). This strongly suggests that over the Phanerozoic, the combination and intersection of multiple different drivers were required to drive substantial variations in climate on 10- to 100-Ma timescales. While these results do not negate the correlations between individual events and climatic excursions, for example, tropical exposure of sutures with global cooling (*9*) or the correspondence of continental arc degassing to the Mesozoic greenhouse (*5*), it does suggest that single processes in isolation are incapable of explaining Earth’s long-term climate state.

### Model limitations

Despite some of the advances that our model can provide, there remain several key limitations that should be addressed in future work. The model uses a single paleogeographic reconstruction and a single series of paleoclimate model runs to inform the climate emulator, meaning the uncertainty in these variables cannot be assessed. Moreover, the model grid times (i.e., times at which paleogeographic maps are used to drive the paleoclimate models) are quite coarse, on average, spanning ~25 Ma, but at times the gap is 40 to 50 Ma. Two consequences of this approach are, first, that many key paleogeographic transitions (such as smaller terranes and blocks entering/leaving tropical zones, emergence of continental shelves, and formation of islands arcs) are missed in our modeling strategy. Machine learning has been used to try to fill these gaps but is not capable of reproducing hydrology to a high standard (*73*). Second, we are unable to easily test alternative paleogeographic configurations, such as Pangea-A versus Pangea B [although see discussion in (*74*) about a resolution to this controversy] or differing locations of terranes in the Permian Meso-Tethyan ocean. A strength of the (py)SCION framework moving forward is that as additional paleoclimate models are generated, they can be incorporated within the climate emulator in the model—irrespective of the spatial resolution—to better resolve these fundamental questions.

It is well established that the FOAM (fast ocean-atmosphere model) general circulation model has a low climate sensitivity (*75*) and, therefore, requires more CO_2_ to increase temperature than more modern climate models. In addition, FOAM has only been run for changes in CO_2_, ignoring other greenhouse gases. This may be why, despite our model predicting temperatures often within the expected ranges (i.e., 15° to 25°C), our *p*CO_2_ estimates are sometimes far higher than the proxy data suggest. The FOAM model also has quite a coarse spatial resolution (7.5° • 4.5°, corresponding to 48 longitudinal cells by 40 latitudinal cells), which is not high enough to properly differentiate finer paleogeographic features such as the opening or closing of oceanic passages. It also struggles to properly capture small-scale drainage systems, such as those that might occur on the seaward side of a continental arc. Consequently, while we consider our model capable of capturing 10- to 100-Ma trends, it is unable to resolve trends on a finer spatial or temporal scale, even if they might be driven by the processes that we model.

A notable biogeochemical limitation of the model is the simple treatment of the organic carbon cycle. Despite implementing a spatially resolved weathering scheme for lithotectonic features and the silicate weathering process, the model implementation of oxidative weathering of sedimentary organic carbon is only related to local runoff, whereas erosion rate and lithology are likely also important (*11*, *76*). Marine organic carbon burial is not calculated spatially despite the geologic record suggesting it is heterogeneous (*77*), and the model also does not feature a spatially resolved dynamic vegetation model for calculating land-derived organic carbon burial. Such a model has been developed for the Mesozoic and Cenozoic and has been shown to alter SCION model predictions (*78*), but it has not yet been developed for the Paleozoic floras. Overall, we consider that a more sophisticated paleogeography, climate emulator, and organic carbon cycle would improve the model and could help further reconcile model results with proxy data during the misfit periods that we note. However, we believe that these advancements are unlikely to reverse the major cooling or warming trends in our results, as the net effect of amplified continental weathering is still likely to be global cooling. Although organic carbon weathering is a CO_2_ source, it is accompanied by nutrient delivery and a reduction in atmospheric O_2_, which both encourage additional organic carbon burial.

We conclude that the prolonged Phanerozoic icehouses during the Carboniferous-Permian and Cenozoic were initiated and maintained through the interaction of multiple “climate drivers” rather than being the result of a single dominant process. This helps to explain why icehouse periods have been relatively rare during Earth history, why so many different drivers have been proposed, and why icehouse-greenhouse transitions have previously proven difficult to reproduce in more simplified carbon cycle models (*3*). Our results suggest that changes in paleogeography, which result in spatio-temporal variations in the hydrological cycle, alongside changes in degassing, are the two most important mechanisms. Continental arc weathering intensity is substantial during warm periods, in agreement with (*8*), but is not able to cause an icehouse transition by itself. Our findings suggest that glacial climates on Earth depend on the interplay between multiple global processes that occur simultaneously, carrying important implications for understanding Earth’s long-term habitability. The long-term temperature of our planet appears to be mechanistically regulated toward an ice-free state rather than a glacial climate.

## MATERIALS AND METHODS

### SCION and pySCION

The model runs were performed using pySCION, a pythonic version of the SCION model (*23*). The underlying SCION model (written in MATLAB) is available at https://github.com/bjwmills/SCION. Then, porting the model to python, the framework is extended to incorporate the impact of different lithologies on the weathering equations. The pySCION model functions as an amalgamation of the COPSE biogeochemical forward model (*27*) and the spatially resolved GEOCLIM model (*24*). Here, the pySCION model uses the data structure of GEOCLIM to resolve key hydrological and climate fields (e.g., runoff and temperature) at each grid time step to calculate erosion and weathering. However, unlike the GEOCLIM model, which is noncontinuous, in between grid time steps, the COPSE biogeochemical model is used to integrate fluxes between time steps. Thus, pySCION can leverage spatially resolved data through time, allowing us to interrogate the effects of long-lived geologic features on the carbon cycle. The model is available as part of the submission in data S2. Full model equations for SCION and pySCION are provided in (*23*). They are also available at the original SCION GitHub repository.

### Arc and suture weathering grids

The same approach was taken to include both continental arcs and ophiolitic-bearing sutures in the model, whereby we calculate the fractional grid area of each cell that was composed of each lithotectonic feature that represent estimates of the spatial extent of intermediate and mafic-ultramafic rocks, respectively. For continental arcs, we used the plate model of Merdith *et al.* (*30*) [the Phanerozoic portion of this model was adopted from models in (*79*–*81*)] to extract all subduction zones within a specific distance of a polygon (representing continental crust) at the pySCION grid times. The distances used were 250 and 436 km, which represent the mean and mean plus one SD distance of arc volcanoes from trenches at the present-day (*82*). We refer to these arcs as “pericontinental” arcs rather than strictly continental arcs because it is possible that our analysis includes some arc systems that may not be geologically classified as continental arcs. For sutures, we used the database of Macdonald *et al.* (*9*), who assembled a record of ophiolite-bearing sutures arising from arc-continent collisions during the Phanerozoic. At each time step, the maps of arcs and sutures were rasterized onto a regularly spaced rectangular grid using the Phanerozoic reconstruction model provided in Macdonald *et al.* (*9*)—a modified version of (*72*, *83*). A particular shortcoming with this approach is translating linear features into area representative domains (as the digitization provided is in the form of polylines). The resolution of the rasterized grid for sutures was 18.75 • 18.75 km, reflecting the scale of the maps they were taken from (1:3,750,000) (*84*), and is slightly finer than estimates of ophiolite thickness from other compilations, where ophiolite sheet width typically ranges between 40 and 60 km (*85*, *86*). For the arc data, we used a coarser grid cell size of 100 km, equivalent to the order of magnitude of width of continental arcs from global compilations and analyses (*7*, *87*). In addition to rasterizing the data to these fine(r) grids, we also rasterize it to the resolution of the pySCION model (48 • 40) (e.g., fig. S3). Thus, at this point of our analysis, we have two grids for each of arcs and sutures (four in total), one representing the resolution of the data and the second at the resolution of the pySCION model.

To determine the fraction of each pySCION grid cell, we use the latitude-longitude bounds of the coarser pySCION resolution map (fig. S3C) to cluster the finer resolution map. For each grid cell at the coarse resolution, we sum the area of corresponding arc or sutures from the finer resolution map to calculate a fraction of the total grid cell area of the coarser grid that is either arc or suture. Because of differences between the underlying paleogeography and the reconstruction used, coupled with the absence of a rotation file of the underlaying SCION paleogeography, some manual manipulation was required to better align the reconstructed sutures and arcs with their position on the land-sea masks.

In cases where sutures overlie arcs or relict arcs, we do not subtract the area contribution attributed to sutures from both arcs and relict arcs, on the basis that sutures are features that are emplaced tectonically onto existing arc systems. Conversely, in situations where arcs overlie relict arcs, we subtract the area of active arcs from relict arcs to prevent double counting these regions. As the resolution of our digitization of ophiolite-bearing sutures is 18.75 km, we multiply their abundance by 3 to better reflect their preserved width [e.g., see compilation by Porkoláb *et al.* (*86*)]. The digitized width of arcs is roughly equivalent to their actual width, so we do not use a multiplier for their abundance.

### Calibrating weathering

As the standard weathering in SCION assumes an average lithology and reactivity across the entire globe, we introduce a weathering factor to represent the enhanced weathering that is expected from silicate rich rocks. We do this by optimizing the parameters in the SCION weathering equations (*40*, *88*) in the modeled 0-Ma timeslice against the present-day measured weathering flux from a series of drainage basins (*40*) using a least-squares optimization from the python library *SciPy*. We include in our modeled weathering flux the distribution of pericontinental arcs and ophiolitic sutures and introduce an AEF and SEF to the weathering equations to isolate what enhancement is necessary to match present-day weathering rates, as measured from dissolved loads in world rivers. We use the same AEF value for our enhancement of relict arcs. The values we let vary, their range, and the results of the optimization are summarized in table S1. Bounds for *K*, *K*_w_, and σ are taken from Maffre *et al.* (*40*).

For the AEF and SEF, we enforce a lower bound of 7 and an upper bound of 20. These enhancements were selected to reflect present-day enhanced weatherability of silicate rocks relative to an average weatherability of continental crust. The lower limit of 7 was established as minimum increased reactivity (*35*). We established an upper limit based on the area of peri-continental arcs and ophiolitic-bearing sutures in our model at present-day, and their total contribution to the silicate weathering cycle. It has been suggested that volcanic silicate weathering accounts for 30% of total weathering at present-day (*22*) on the basis of ^87^Sr/^86^Sr and ^187^Os/^188^Os curves. In our analysis, the combined present-day area of pericontinental arcs and ophiolitic bearing sutures is 1.5% of Earth’s surface area. Thus, we infer that a 20-fold weathering enhancement is a reasonable upper limit.

To assess our optimized values for arc and sutures factors (in addition to the other parameters), we plot them against measured data from present-day (*40*) (fig. S3, E to G). We find that our optimized enhancement factors produce a better fit to present-day riverine flux (fig. S3G) than the unoptimized run with no enhanced weathering (fig. S3F). In particular, there are no outliers that overestimate weathering, indicating that our results (and weathering enhancement factors) are likely to be conservative.

To implement these grids within the pySCION framework, we multiply the fractional content of each land cell that is “ARC,” “RELICT ARC,” or “SUTURE” according to the appropriate weathering multiplier (that is, AEF and SEF) using the following equations

$$\omega_{\text{arc}}=\omega_{\text{silw}}\times \;\left[1+K_{\text{arcfrac}}\times \left(\text{AEF}-1\right)\right]$$ (1)

$$\omega_{\text{relictarc}}=\omega_{\text{silw}}\times \;\left[1+K_{\text{relictarcfrac}}\times \left(\text{AEF}-1\right)\right]$$ (2)

$$\omega_{\text{suture}}=\omega_{\text{silw}}\times \;\left[1+K_{\text{suturefrac}}\times \left(\text{SEF}-1\right)\right]$$ (3)

These equations are then combined as

$$\omega_{\text{totalsilw}}=\omega_{\text{silw}}+\omega_{\text{arc}}+\omega_{\text{relictarc}}+\omega_{\text{suture}}$$ (4)

### Degassing

Previous models, including COPSE (*27*) and GEOCARB(SULF) (*89*), invert Phanerozoic sea level to approximate crustal production at mid-ocean ridges and then use that measure as a proxy for degassing (*89*, *90*) (i.e., CO_2_ output from the lithosphere into the atmosphere). To quantify degassing in our model, we divide it into five different tectonically driven sources (Fig. 1A): (i) continental rifts; (ii to iv) convergent margins, including carbon sourced from the (ii) subduction of carbonaceous ocean sediments, (iii) degassed from the magma chambers (either actively or passively), or (iv) degassed diffusively on the flanks of volcanic arcs; and (v) mid-ocean ridges. Our analysis assumes that the partitioning of carbon from solid earth processes within these environments is linear through time—the same proportions of carbon are being emitted now as in the past, and the only factor changing this amount is variation in tectonic fluxes. Our approach is therefore to treat each source of degassing separately, determining first an appropriate present-day flux, then deriving a scaling factor able to scale the present-day flux back in time, and, lastly, combining the scaled measurements into a single “total” degassing curve. We use the 10th and 90th percentiles of the present-day flux to divide the minimum and maximum estimates to express the degassing curve as a flux relative to present-day. When we fully preserved the present-day uncertainty (i.e., the present-day ratio of the compiled curve is the same as the present-day relative curve), our uncertainty envelope encompassed all temperatures between 15° and 25°C over the Phanerozoic.

Our final flux is assembled from the raw data before being smoothed using a Savitzky-Golay filter with a 21-Ma moving window and polynomial order 1. Data S1 provides the data we used. We selected a 21-Ma moving window as this provided a good balance to smoothing short term perturbations that could be artifacts in the underlying plate-tectonic model while still preserving variations on a 10-Ma scale.

### Scaling mid-ocean ridge processes

To scale the processes occurring at mid-ocean ridges, we first derive a tectonic forcing curve constrained by three (I to III) separate curves estimating a gross tectonic flux at ridges and/or subduction zones. We use the range covered by all three curves to accommodate uncertainty in gross estimates of pre-Pangea subduction and spreading flux. (I) A recent study (*17*) calculated a tectonic degassing curve back to 550 Ma. They used a hybrid approach of crustal consumption curves calculated from full-plate reconstructions (*91*) for the Mesozoic-Cenozoic and zircon data for the Paleozoic (*80*, *92*) (where the zircon flux is proposed as a proxy for subduction flux). For our mid-ocean ridge, we use their hybrid curve (derived from zircons and full-plate models). Our second and third estimates of tectonic flux use crustal consumption (II) and production (III) curves from a plate model (*30*). We assume that over 1- to 10-Ma timescales crustal production (i.e., area of new seafloor created at mid-ocean ridges) is equivalent to crustal consumption (i.e., area of seafloor consumed at subduction zones), which can be demonstrated quite for the past 150 Ma (*6*), but before this time, there are differences between the two curves. We extract both crustal production and consumption curves from 550 to 0 Ma. To create our final tectonic forcing curve that encompasses all three individual curves, we take the group minimum and maximum across all three curves at each time step.

### Scaling continental arc processes

To scale the processes occurring at continental arcs, we adopt a similar approach to our method for mid-ocean ridges. We constrain continental arc flux by three separate curves (I to III), one derived from zircon spectra (*17*) and the second and third from the subduction flux extracted only from pericontinental subduction zones using two different distance thresholds. We deliberately refer to these arcs as pericontinental, rather than continental arcs, as limitations within plate models (e.g., rigid polygons and uncertainty about extent of continental crust back in time) make it an imperfect estimate of “true” continental arc length. As with the mid-ocean ridge flux, we use the range covered by all three curves to accommodate uncertainty in gross estimates of continental arc flux. (I) The study of Marcilly *et al.* (*17*) also produced a degassing estimate derived completely from zircon. As their curve is explicitly defined from arc-derived zircon, we take this as a direct proxy for continental arc flux through time. Our second and third estimates of tectonic flux use crustal consumption curves from the plate model (*30*). However, this time we filter the subduction zones in the model by their distance to the nearest polygon in the plate model to extract those that could plausibly be a continental arc (rather than an oceanic arc), in the same manner we applied to produce the continental arc silicate weathering grids. We used two distance thresholds (*82*), representing the (II) mean distance (250 km) and (III) mean distance plus one SD (436 km) from a modeled subduction zone to volcanic arc at present day. We then multiplied these lengths by the time-sensitive, average convergence rate extracted from the plate model (*30*) to determine an estimate of minimum and maximum pericontinental arc flux. To create our best estimate of continental arc flux curve that encompasses all three individual curves, we take the group minimum and maximum across three at for each time step.

### Rifts

CO_2_ can be degassed diffusely from active continental rifts by mobilization of subcrustal reservoirs and migration via extensive fault and fracture networks (*93*, *94*). There is a large range in degassing measurements across rift environments (*95*), with fluxes from the Madagi-Natron Basin in East African Rift (EAR) system thought to possibly contribute up to ~1 Mt C/a (*93*), while the back-arc Taupo Volcanic Zone in New Zealand (which occupies a similar area to the Madagi-Natron Basin) contributes an order of magnitude less, ~0.1 Mt C/a (*96*). Propagating fluxes along the entire EAR lead Lee *et al.* (*93*) to propose a total rift degassing of 14 to 26 Mt C/a, roughly equivalent to degassing from the entire mid-ocean ridge system, although this range was re-evaluated by Hunt *et al.* (*94*) to a more conservative 1 to 8 Mt C/a. Werner *et al.* (*95*) suggested fluxes from other rifts could potentially add another 8 to 11 Mt C/a, for a total global flux of 13 to 16 Mt C/a [using the mean EAR value in (*94*)], although cautioned that many more measurements are needed. Last, Brune *et al.* (*97*) compiled a database of rift lengths for the Mesozoic-Cenozoic and estimated global fluxes of CO_2_ from rifts at present-day. They proposed two estimates of total contribution to C from present-day rifts, a mean estimate of 54 Mt of C/a, and a conservative estimate of 13.5 Mt of C/a. The primary difference of the two measurements reflects uncertainty around the large fluxes coming from parts of the EAR relative to other rifts. As the conservative estimate of Brune *et al.* (*97*) falls reasonably well in the center of the tentatively proposed global range of Werner *et al.* (*95*), we use their range as a conservative uncertainty estimate of rift degassing.

To scale this present-day value back through time, we use a rift-length database (*98*), as organized and processed in Brune *et al.* (*97*) (450 to 0 Ma) and extended by Merdith *et al.* (*99*) (450 to 1000 Ma). However, one issue with the transition between the two databases is that the Neoproterozoic and Early Paleozoic database predominantly identifies rifts that form during continental breakup and/or terrane detachment and migration. Comparatively, the database in (*98*) contains not only these rifts but also rifts formed in convergent settings, such as back-arc basins. This is likely a function of preservation bias, and to counteract this issue, we calculated the fraction of rifts formed in convergent settings (*98*), which for the period 450 to 0 Ma is 0.44 [i.e., on average, 44% of total rift length in the database (*98*) formed in convergent settings and is unaccounted for in the estimate in (*99*)]. Assuming that this proportion is constant back to 600 Ma, we applied a rift length multiplier

$${\text{length}}_{\text{adjusted}}=\text{length}\times {\left(1-0.56\right)}^{-1}$$

to the rift lengths in (*99*).

Last, the rift database (*98*) only provides start times of each rift in the form of a geologic time period or epoch, and so an implicit assumption of our quantification is that the rifts were only active for the duration bounded by their identified geologic timescale age. This creates an issue for very young rifts, initiated since the Neogene, as their duration age (i.e., time we consider them “active” and degassing) becomes very short relative to older rifts that might have been attributed a start age of a longer epoch. Our estimate of rift lengths drops quite abruptly from the Pliocene to present-day (~25,000 to ~8000 km) because rifts given ages of “Pliocene” or “Pleistocene,” for example, do not contribute to the present-day estimate. Therefore, when we smooth our degassing curve, we do not fix the present-day estimate, and the result is that the smoothed curve increases at present-day and reduces the influence of rifts back through time.

### Peri-continental arc derived carbon

Much work was been done on disentangling and isolating different sources to solid-Earth degassing from continental arcs (*4*, *100*, *101*). Typically, the breakdown of continental arc carbon degassing is done on the basis of temperature, separating deeply sourced magmatic carbon (>450°C) from more shallow hydrothermally derived carbon (*102*–*104*). Here, we separate carbon sourced from continental arcs into two different categories: magmatic carbon sourced deeply from either the (ii) subducting sediments or (iii) continental crust and (iv) diffuse degassing along continental arcs (such as through soils and springs).

### Magmatic degassing

Within subduction zones, slab-derived carbon is liberated due to the warming and devolatilization of the slab and/or sediments being subducted and liberation of carbon stored in lower crustal reservoirs (*105*). Carbon pathways in subduction zones are poorly constrained, and most of the estimates of bulk carbon fluxes from these sources are based on mass-balance estimates between carbon entering a subduction zone and what is degassed (*4*, *100*). In addition to improvements in observation of volcanic degassing over the past 20 years [e.g. (*104*)], advancements have also involved identifying isotopic signatures of different sources (*106*, *107*). Analyzing data from a global compilation of fluxes, Aiuppa *et al.* (*106*) isolated high temperature volcanic degassing. They argue that based on the S/C and Ba/La ratios, and LILE enrichment patterns of volcanic arcs, that most of the carbon emitted from these volcanoes come from the subducting slab (68 to 81%), particularly, from the sediments of the subducting slab. The remaining 19 to 31% of magmatic global volcanic arc emissions are thought to be sourced from assimilation of carbon reservoirs in the lower crustal column [e.g., arc magma-limestone interaction; (*105*)] based on their heavy enrichment in LILEs.

Volk (*108*) first speculated that an increase in carbon degassing through the Cretaceous present-day must have been driven by an increase in deep-ocean carbon burial [see also *109*)]. This hypothesis has been part of all biogeochemical models since the original GEOCARB and COPSE models (*21*, *29*), typically referred to as the “B-factor” (for carbon burial), and scale down degassing before 140 Ma to 75% of present-day. Müller *et al.* (*6*) provided thermodynamic modeling of carbon pathways to estimate the volume of carbon sourced from subducting slabs over the Mesozoic and Cenozoic and argued that most of slab-derived carbon comes from subducting sediments (total influx into overriding plate at present-day of 31 to 70 Mt C/a). This study, which focused on the oceanic conveyer belt component of the deep carbon cycle, did not consider overriding crustal sources of carbon, and the atmospheric outflux (i.e., a mass balance approach between inputs into the overriding plate and outputs measured along volcanic arcs) was calibrated by Bekaert *et al.* (*110*), who estimated 14 to 24 Mt C/a based on carbon/sulfur (C/S) ratios from (*103*). Müller *et al.* (*6*) ascribed the entire reservoir from subducted sediments to this amount being outgassed; however, here, as we also consider assimilated CO_2_ from overriding carbon storage, we partition this total amount into two sources, 68 to 81% from the sediment estimation of Müller *et al.* (*6*) and the remainder from the overriding plate. In effect, we take the exact outgassing flux of Muller *et al.* from 250 to 0 Ma and scale it down to 68 to 81% [i.e., (ii) 10 to 19 Mt C/a at present-day]. This represents the component of carbon sourced from pelagic sediments and replaces the B-factor in our model. Second, using the present-day estimate of 14 to 24 Mt C/a, we take the remainder 19 to 32% as the present-day flux of carbonate assimilation in the overriding plate [(iii) 2 to 7 Mt C/a]. This later range is scaled back in time through our pericontinental arc flux curve compiled above.

### Diffuse or passive degassing along continental arcs

The contribution of diffuse degassing along continental arcs—such as CO_2_ released through soils, spring lakes, and vents on the flanks of volcanoes—is thought to be a large, but uncertain, contribution to tectonic degassing. We use the present-day estimates of a recent compilation (*104*), which suggest 14 ± 1.5 Mt C/a is released. This estimate only pertains to diffuse, low-temperature degassing on continents and does not include contributions from other submarine sources [e.g., (*111*)]. We use our pericontinental arc flux to scale these values back in time (fig. S5).

### Mid-ocean ridges

Mid-ocean ridge degassing is driven by the release of CO_2_, and other volatiles, from the mantle beneath a spreading ridge. In these environments, the amount of CO_2_ released during the formation of newly formed oceanic crust is typically calculated by analyzing the ratio of CO_2_ to another element, chiefly ^3^He or Ba (*112*). Most estimates using these methods place the present-day flux at mid-ocean ridges between 10 and 100 Mt C year^−1^ [e.g., (*113*)], corresponding to a mantle concentration of around 100 ppm. Keller *et al.* (*114*) used a geodynamic model to calculate the spreading rate-dependent degassing of mid-ocean ridges, given a mantle concentration of C. This was used to estimate (*6*) carbon degassing at mid-ocean ridges over the past 250 Ma, with a resultant present-day flux of 13 ± 4 Mt C year^−1^, using a mantle carbon concentration of 100 ± 20 ppm [e.g., (*115*)]. In our analysis, we use the present-day derived estimate of Müller *et al.* (*6*) and compile a composite tectonic-forcing curve back to 550 Ma to scale the relative MOR degassing back in time. This is a simplification of the method of Müller *et al.* (*6*) who calculated the flux dynamically based on known spreading rates at known mid-ocean ridges (*114*). However, the bulk of our analysis covers the time interval pre-Pangea breakup, thus making it impossible to use the same method unilaterally with the same degree of confidence. Instead, we scaled the present-day estimate of Müller *et al.* (*6*) using our tectonic forcing curve.

### Degassing of the Siberian Traps and other (s)LIPs

To investigate how the rapid release of large amounts of lithospheric carbon via large igneous provinces (LIPs) may affect our model, we performed a model run including estimates of LIP degassing (a table this data is included in the supplementary model files). Our model run is a nonexhaustive compilation of Phanerozoic LIPs (*35*) but does include key events including the Siberian Traps, Central Atlantic Magmatic Province, Ferrar-Karoo, and Deccan Traps. We also include the Whitsunday Volcanic Province, a ca. ~15-Ma long-lived sLIP that formed in the Cretaceous on the eastern margin of Australia (*116*). The results of this model run are shown in fig. S14. We found that LIPs do not perturb the model climate on >10 Ma timeframes—which are the focus of this study. This is because LIPs are either shorter lived than 10 Ma, or if they do have longer activities (e.g., Whitsunday Province), then this leads to a lower rate of CO_2_ injection. Some LIPs do perturb the model climate on <10 Ma timescales, and the Siberian Traps causes a multidegree warming over several million years in our model. This is because carbon release rate from this LIP is thought to be up to an order of magnitude higher than other LIPs, up to 1 × 10^13^ mol C/a (*38*) over ~2 Ma. For this reason, we include that the Siberian Traps in our default model runs as an endmember (maximal) example. Our analysis does not preclude LIPs acting as important climate drivers on shorter timescales (e.g., through pulsed degassing), but these events are not the focus of this work.

The implementation of these LIPs into pySCION is done in a similar manner to previous work (*117*), where we include LIP degassing as a forcing that is described by a Gaussian function, with the midpoint defined halfway through the LIPs duration and the width set to half the duration time. The calculated *p*CO_2_ contribution from this Gaussian is then added to the degassing flux. We assume this carbon has the same isotopic composition (−5‰) and model the carbon mass balance as such.

### Isolating mechanisms

To test the influence and strength of individual mechanisms, we isolate their contributions to be consistent with what is observed at present-day.

### Sutures, arcs, and degassing

For degassing, we assign a value of ‘1’ for each time step, thus ensuring that degassing remains constant through time at the present-day value. For sutures and arcs, we simply set their enhancement factors to 1, so that they are considered to have the average weatherability of continental crust (equivalent to the default SCION model).

### Paleogeography

Isolating the effects of paleogeography are more difficult and to properly test this would require alternative paleogeographic (and their dependent paleoclimate) models. As a simple measure to test the effect of paleogeography on hydrology and weathering, we alter the values of the slope and runoff to be equal to the mean value of the globe at each time step and CO_2_ step. This acts to reduce any change in gross silicate weathering but instead spreads it across the entire land mask at any time step (rather than being concentrated in certain areas). The homogenization occurs before the calculation of any contribution of arcs, sutures, or relict arcs to the silicate weathering flux.
