# Supplementary material for: Phanerozoic icehouse climates as the result of multiple solid-Earth cooling mechanisms
Source: Sci Adv. 2025 Feb 14;11(7):eadm9798. doi: 10.1126/sciadv.adm9798 (PMC11827867; doi:10.1126/sciadv.adm9798)
Supplement: Supplementary file 1 — Figs. S1 to S15 Table S1 Legends for data S1 and S2 [file sciadv.adm9798_sm.pdf]

Science Advances
AAAS

Supplementary Materials for

Phanerozoic icehouse climates as the result of multiple solid-Earth cooling mechanisms

Andrew S. Merdith *et al.*

Corresponding author: Andrew S. Merdith, andrew.merdith@adelaide.edu.au

Sci. Adv. **11**, eadm9798 (2025)
DOI: 10.1126/sciadv.adm9798

The PDF file includes:

Figs. S1 to S15
Table S1
Legends for data S1 and S2

Other Supplementary Material for this manuscript includes the following:

Data S1 and S2

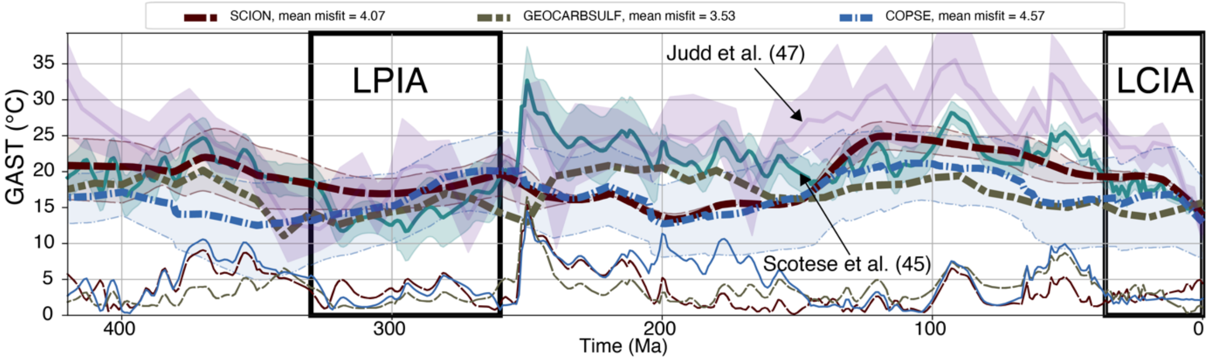

***fig. S1. Global average surface temperature prediction of commonly used biogeochemical models (SCION** (23)**, GEOCARBSULF** (39)**, COPSE** (27)**).** *Misfit refers to the Wasserstein distance (see methods) between the distribution of each result and the temperature of* (45)*. These misfits are shown as the thin lines towards the bottom of the figure. LPIA: late Paleozoic ice age; LICA: late Cenozoic ice age. Solid green line with envelope is the GAST curve of* (45)*, indigo line with envelope is the GAST curve of* (47)*.*

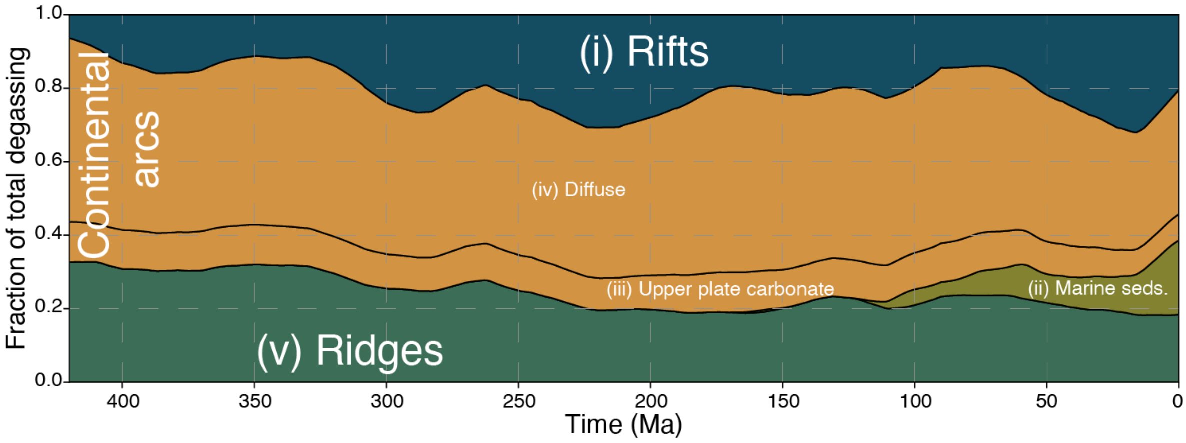

fig S2: Fractional content of total degassing through time from each different source. 'Ridges' refers to mantle degassing along mid-ocean ridges; 'Marine sediments' refers to carbon derived from deep-sea pelagic sediments subducted since the mid-Cretaceous; 'diffuse' refers to low-temperature diffuse degassing around continental arcs (*107*); 'rifts' refers to continental rifts (*98*); 'Deep cont. carbon' refers to carbon assimilated from deep crustal sources around continental arcs (*109*), and 'Shallow cont. seds.' refer to shallow sediments around continental arcs (*126*).

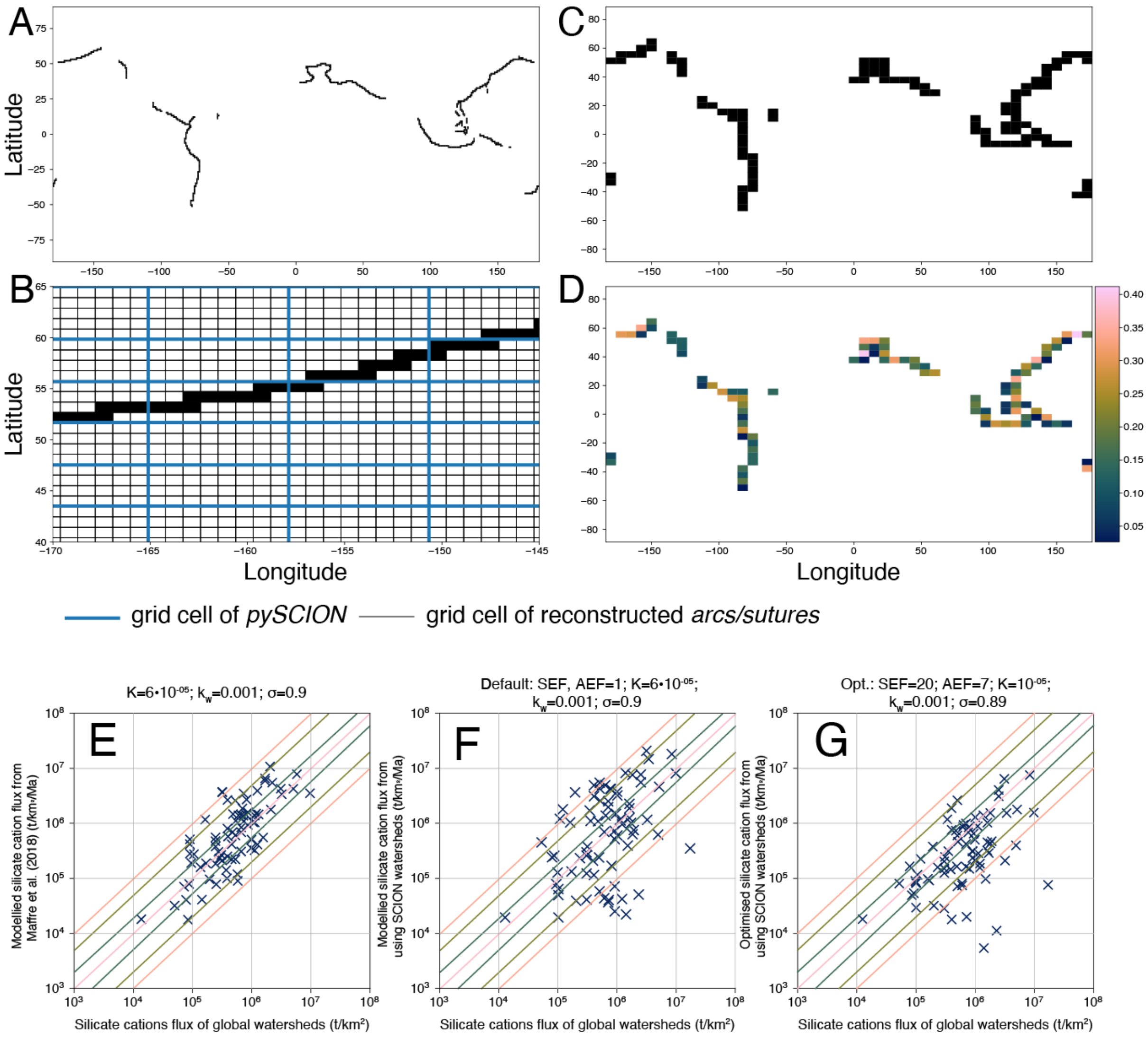

fig S3. Summary of digitization, incorporation and calibration of arcs and ophiolite-bearing sutures and their enhanced weathering factors into *pySCION*. This figure uses maps of peri-continental subduction zones extracted from (*30*) at present-day as an example. (A) Digitization of arcs at original resolution (onto a grid spacing of 100 • 100 km). (B) Digitization of arcs at *pySCION* resolution (onto a grid cell resolution of 7.5° • 4.5°, corresponding to a 48 • 40 grid). (C) Overlay of *pySCION* grid cells (B) above original resolution grid cells (A). For each *pySCION* grid cell (blue lines) we sum the area of arc grid cells in the original resolution, then express this as a fraction of total *pySCION* grid cell area (grid cell area for both resolutions is corrected for latitude). (D) Represents the area-corrected grid cell fraction of continental arcs. Scatterplots showing modeled riverine silicate cation flux compared to what is measured in present-day watersheds, with different erosion and enhancement factors highlighted in the titles. Each point (X) represents a unique watershed. Pink, green, olive and salmon lines represent the 1:1 equivalence line and the 2-fold, 5-fold and 10-fold difference lines, respectively. (E) The 'best-fit' erosion parameters from (*40*). (F) Unoptimized and default values in the *SCION* model. (G) Optimized values, including variations in arc and suture enhancement factors from *pySCION*.

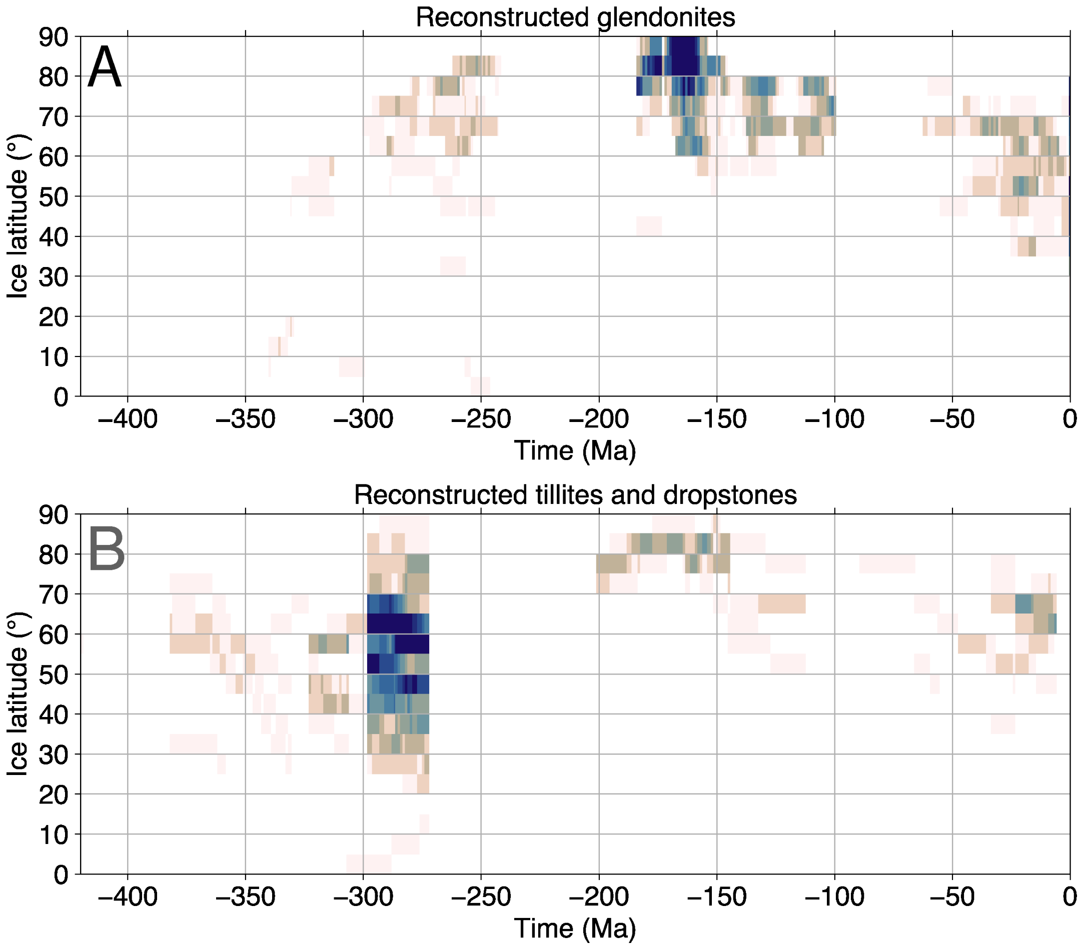

fig S4: Reconstructed lithological data used to constrain paleoiceline. Here we split the dataset into its two components: (A) reconstructed glendonites after Rogov et al. (*43*), and (B) reconstructed tillites and dropstones after Boucot et al. (*42*).

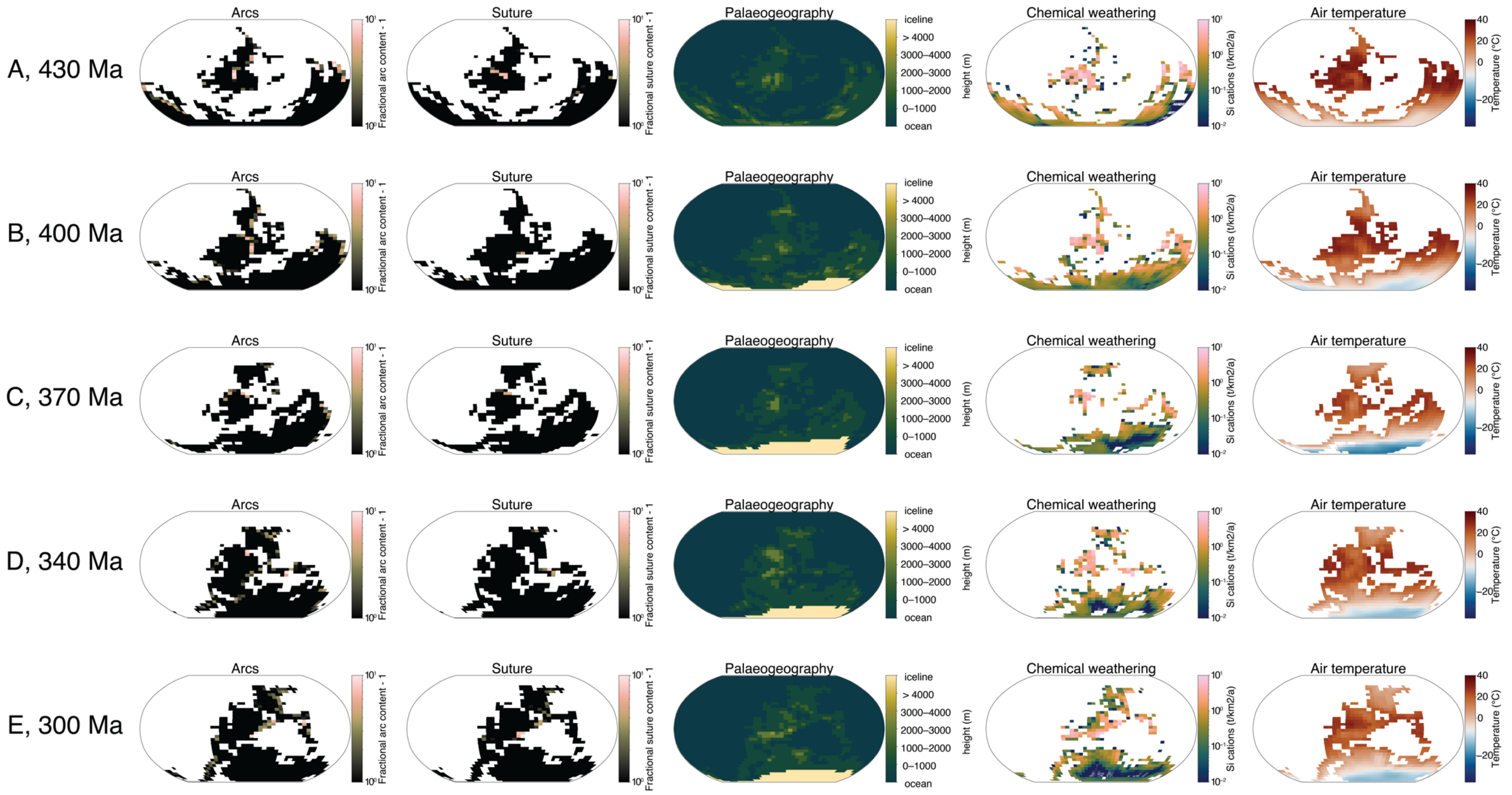

A, 430 Ma
B, 400 Ma
C, 370 Ma
D, 340 Ma
E, 300 Ma
Arcs
Fractional arc content - 1
Suture
Fractional suture content - 1
Palaeogeography
iceline
> 4000
3000–4000
2000–3000
1000–2000
0–1000
ocean
height (m)
Chemical weathering
Si cations (t/km2/a)
Air temperature
Temperature (°C)
40
20
0
−20

fig S5: Mean spatial inputs and outputs from *pySCION* default run (all drivers on). Organized by grid-timesteps from 430 to 300 Ma as rows with variables as columns. Results at (A) 430 Ma, (B) 400 Ma, (C) 370 Ma, (D) 340 Ma, and (E) 300 Ma. Arcs represent locations and fractional content of peri-continental arcs (subtract 1 from grid-cell value to get fractional content). Sutures represent locations and fractional content of ophiolite-bearing sutures after (*9*) (subtract 1 from grid-cell value to get fractional content). Paleogeography depicts underlaying land-sea masks and paleotopography used as boundary conditions for both the FOAM climate models that underpin *pySCION* and the spatial parameters that are used to compute runoff and erosion. Chemical weathering represents the sum of all chemical weathering sources (including arcs and sutures). Air temperature is modelled temperature based on calculated pCO_2 level. Note that the iceline is set at -10°C, while colour scale the inflection point is 0°C.

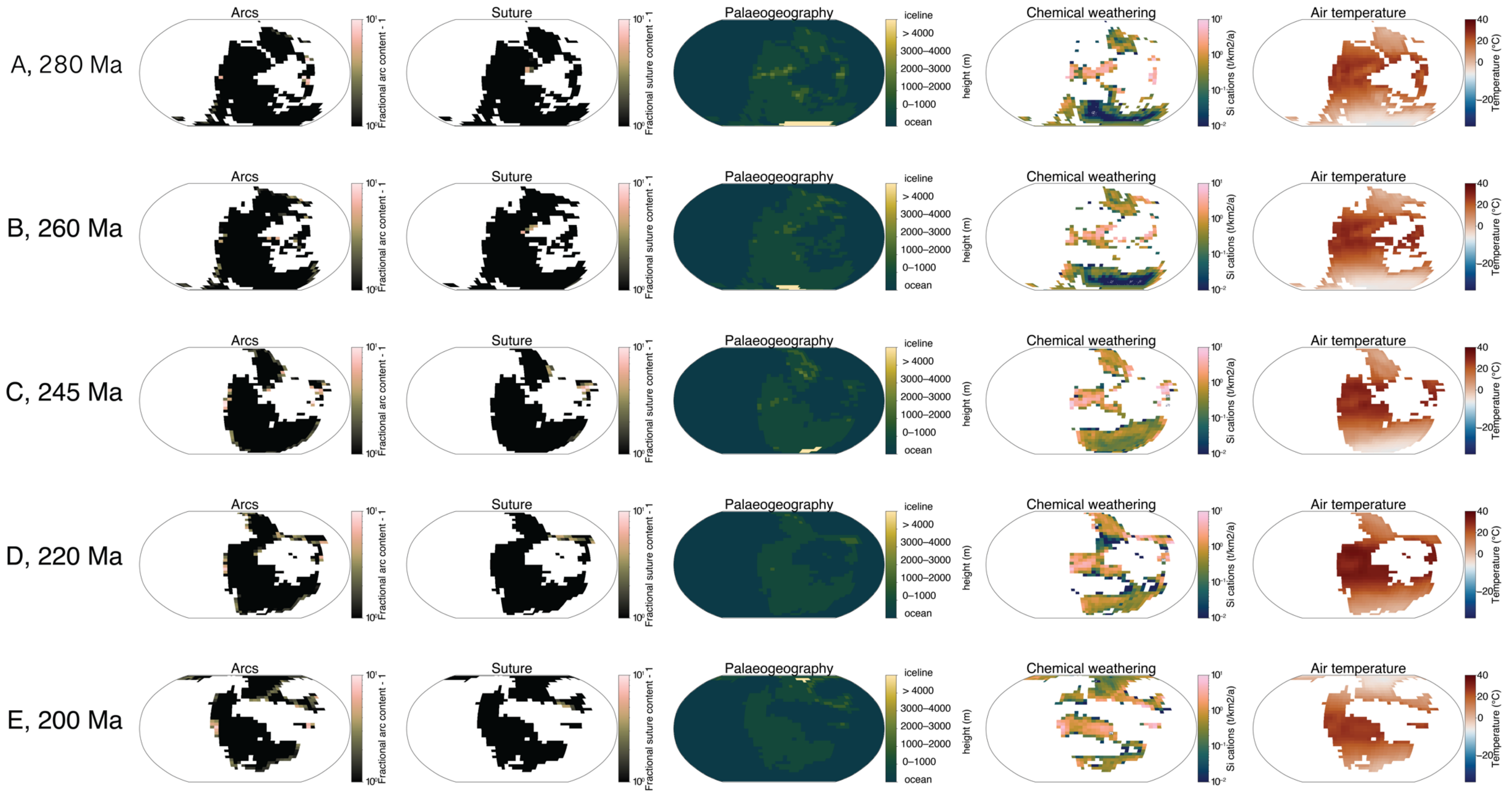

A, 280 Ma
B, 260 Ma
C, 245 Ma
D, 220 Ma
E, 200 Ma
Arcs
Fractional arc content - 1
Suture
Fractional suture content - 1
Palaeogeography
iceline
> 4000
3000–4000
2000–3000
1000–2000
0–1000
ocean
height (m)
Chemical weathering
Si cations (t/km2/a)
Air temperature
Temperature (°C)
40
20
0
−20

fig S6: Mean spatial inputs and outputs from *pySCION* default run (all drivers on). Organized by grid-timesteps from 280 to 200 Ma as rows with variables as columns (as with fig. S5). Results at (A) 280 Ma, (B) 260 Ma, (C) 245 Ma, (D) 220 Ma, and (E) 200 Ma.

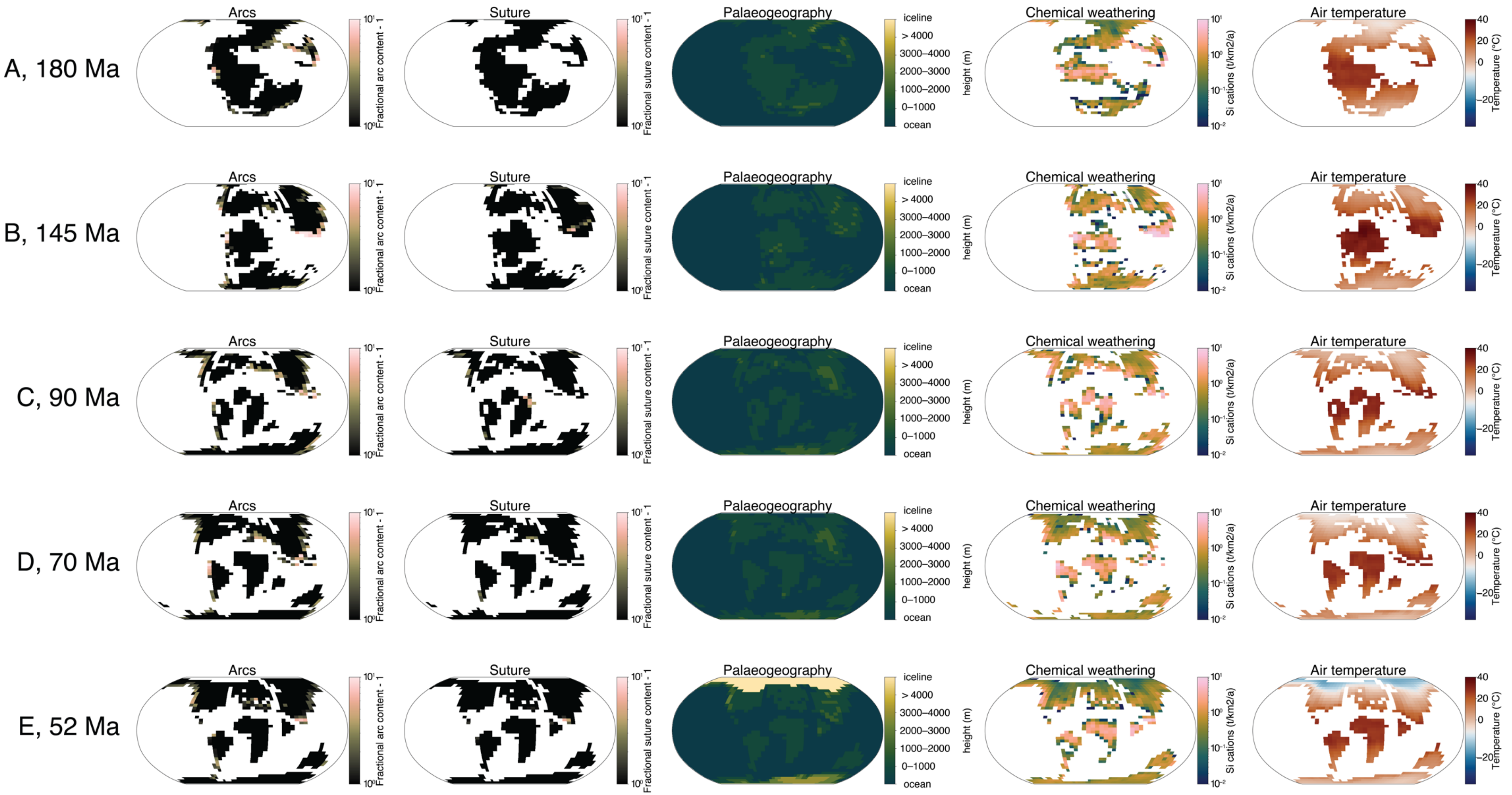
A, 180 Ma
B, 145 Ma
C, 90 Ma
D, 70 Ma
E, 52 Ma
Arcs
Fractional arc content - 1
Suture
Fractional suture content - 1
Palaeogeography
iceline
> 4000
3000–4000
2000–3000
1000–2000
0–1000
ocean
height (m)
Chemical weathering
Si cations (t/km2/a)
Air temperature
Temperature (°C)
40
20
0
−20

fig S7: Mean spatial inputs and outputs from *pySCION* default run (all drivers on). Organized by grid-timesteps from 180 to 52 Ma as rows with variables as columns (as with fig. S5). Results at (A) 180 Ma, (B) 145 Ma, (C) 90 Ma, (D) 70 Ma, and (E) 52 Ma.

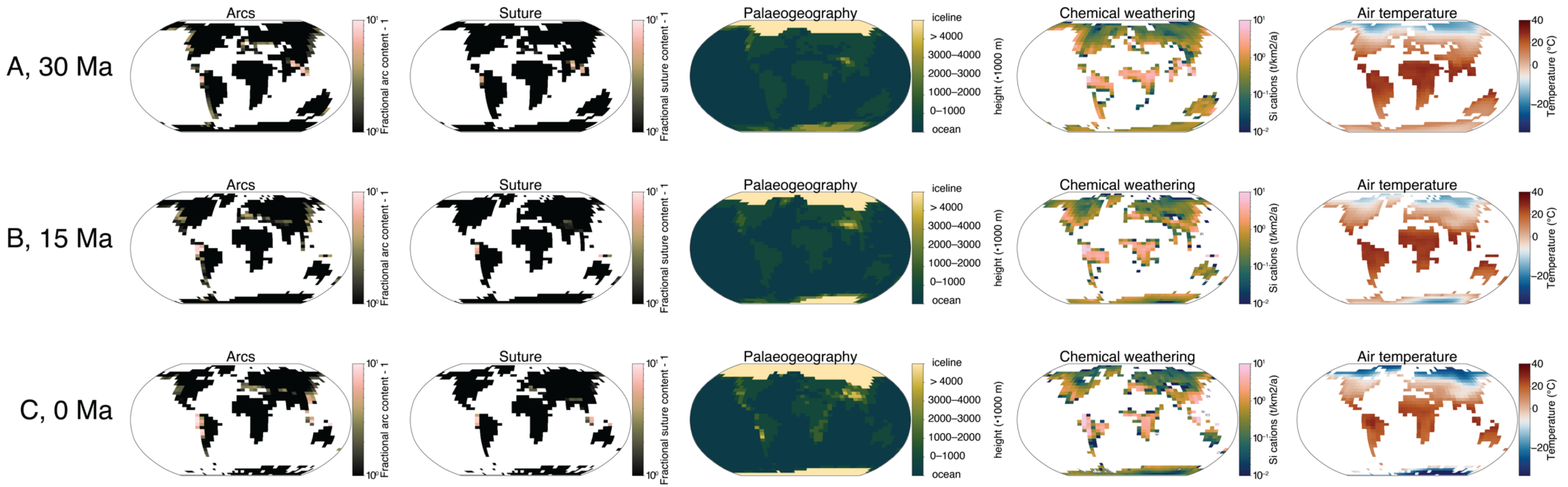
A, 30 Ma
B, 15 Ma
C, 0 Ma
Arcs
Fractional arc content - 1
Suture
Fractional suture content - 1
Palaeogeography
iceline
> 4000
3000–4000
2000–3000
1000–2000
0–1000
ocean
height (•1000 m)
Chemical weathering
Si cations (t/km2/a)
Air temperature
Temperature (°C)
40
20
0
−20

fig. S8: Mean spatial inputs and outputs from *pySCION* default run (all drivers on). Organized by grid-timesteps from 30 to 0 Ma as rows with variables as columns (as with fig. S5). Results at (A) 30 Ma, (B) 15 Ma, and (C) 0 Ma.

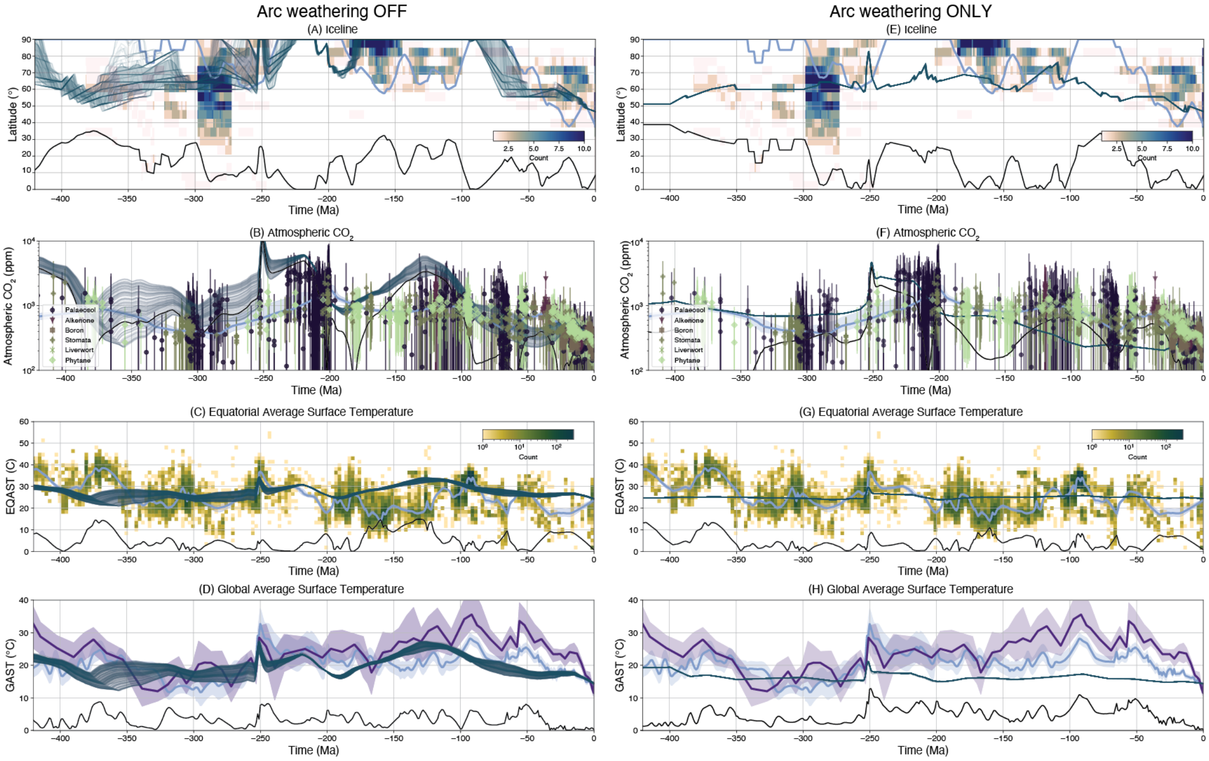

fig. S9: Model run with contribution to silicate weathering cycle from peri-continental arcs and relict arcs 'off' (i.e., no extra contribution) in column 1, and with only their contribution on in column 2. (A and E) iceline extent, with proxy data (*42*, *43*). (B and F) *p*CO_2 levels with proxy data (*57*, *58*). Equatorial average surface temperature from $\delta^{18}O$ (*51*). (D and H) global average surface temperature, against proxy curves of (*45*) (blue) and (*47*) (indigo).

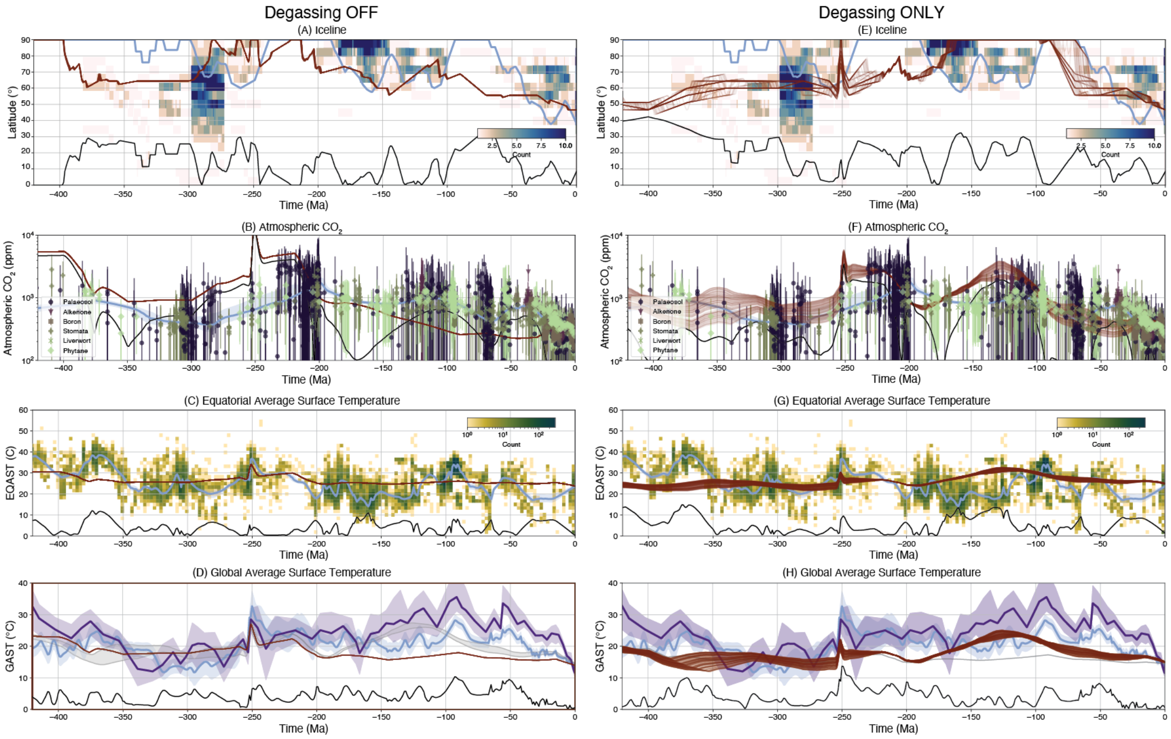

fig. S10: Model run with degassing turned 'off' (i.e., set to present-day levels) in column 1, and with only their contribution on in column 2. (A and E) iceline extent, with proxy data (*42*, *43*). (B and F) pCO_2 levels with proxy data (*57*, *58*). Equatorial average surface temperature from $\delta^{18}O$ (*51*). (D and H) global average surface temperature, against proxy curves of (*45*) (blue) and (*47*) (indigo).

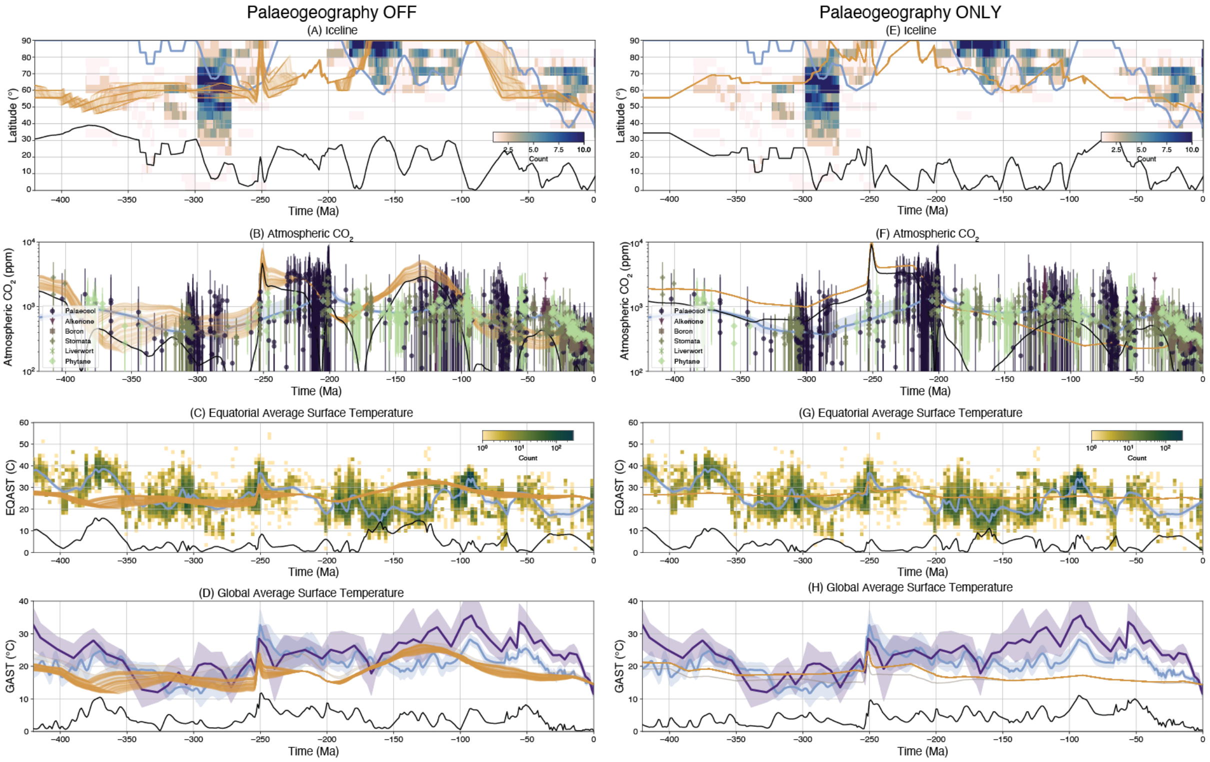

fig. S11: Model run with paleogeographic variation turned 'off' in column 1, and with only their contribution on in column 2. (A and E) iceline extent, with proxy data (*42*, *43*). (B and F) $p\text{CO}_2$ levels with proxy data (*57*, *58*). Equatorial average surface temperature from δ^{18}O (*51*). (D and H) global average surface temperature, against proxy curves of (*45*) (blue) and (*47*) (indigo).

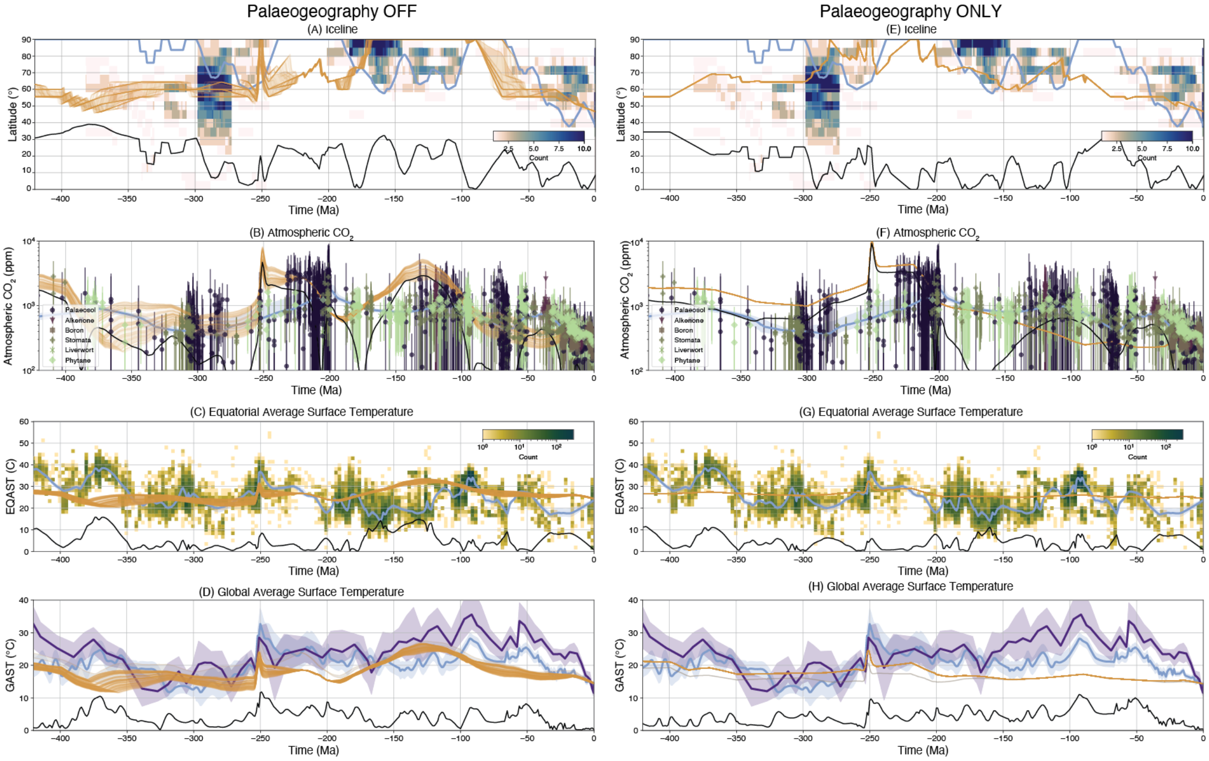

fig. S12: Model run with contribution to silicate weathering cycle from ophiolite-bearing sutures 'off' (i.e., no extra contribution) in column 1, and with only their contribution on in column 2. (A and E) iceline extent, with proxy data (*42*, *43*). (B and F) *p*CO_2 levels with proxy data (*57*, *58*). Equatorial average surface temperature from $\delta^{18}O$ (*51*). (D and H) global average surface temperature, against proxy curves of (*45*) (blue) and (*47*) (indigo).

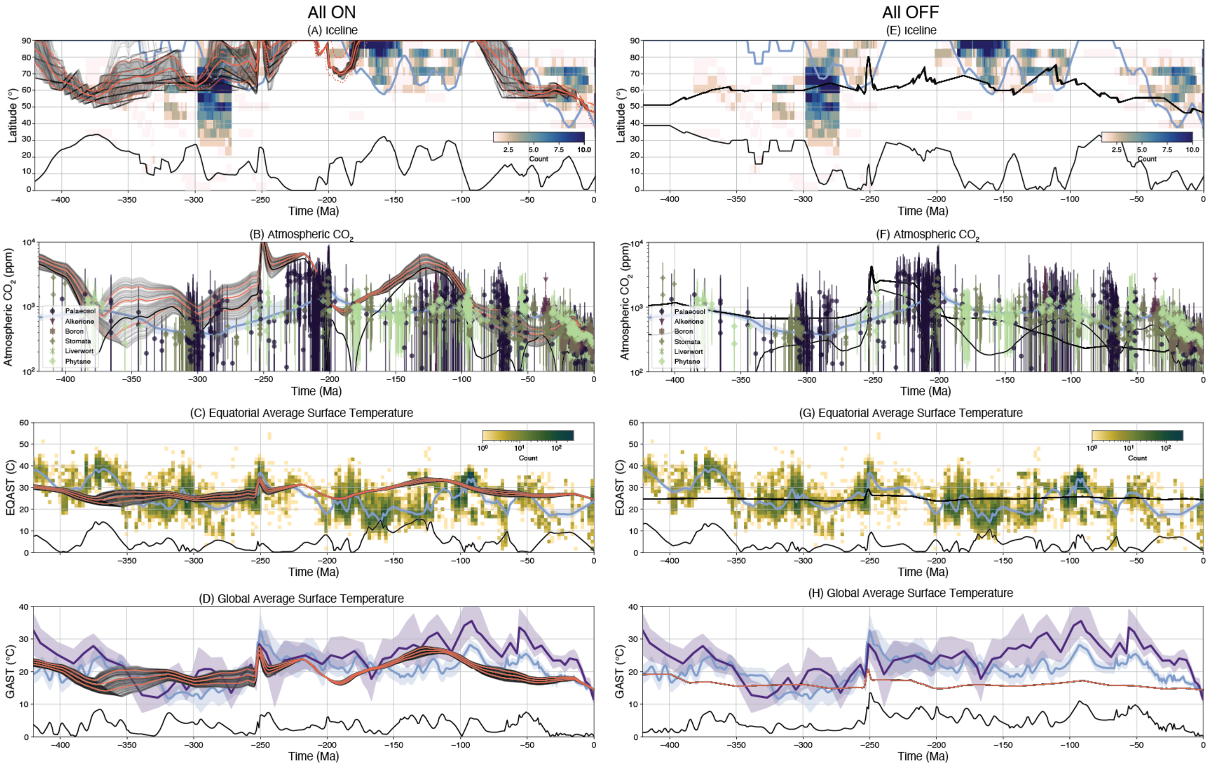

fig. S13: All drivers on (column 1, equivalent to Fig. 2 in main text) and off. (A and E) iceline extent, with proxy data(*42*, *43*). (B and F) pCO_2 levels with proxy data (*57*, *58*). Equatorial average surface temperature from $\delta^{18}O$ (*51*). (D and H) global average surface temperature, against proxy curves of (*45*) (blue) and (*47*) (indigo).

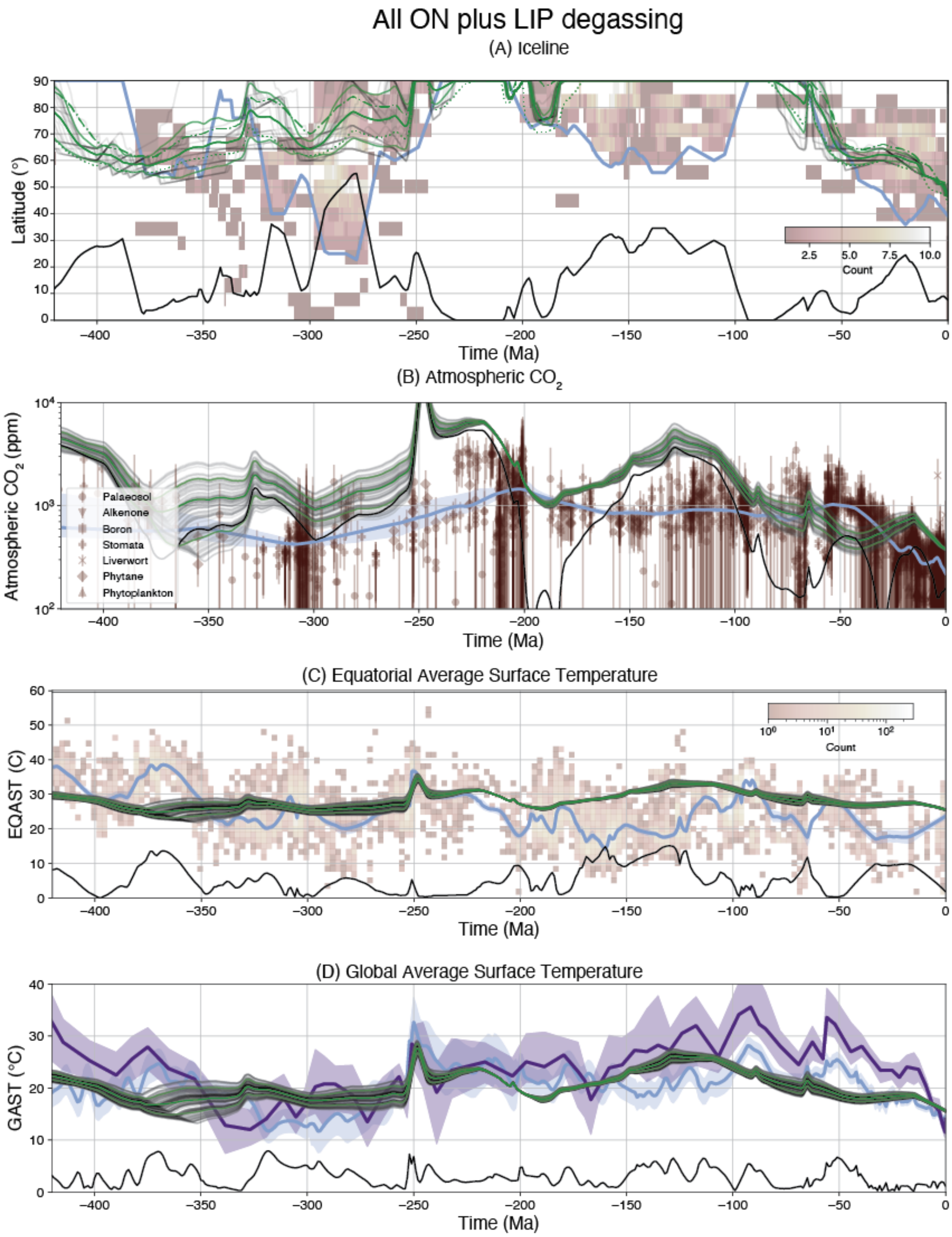

fig. S14: 'Default' all drivers on model run including estimated LIP degassing after (*35*). We also include estimated sLIP degassing of the Whitsunday Volcanic Province of ~1.67 • 10^{11} moles C/a, assuming a LIP volume of 2.5 •10^6 km^3, a degassing duration from 120–105 Ma and a similar degassing proportion of the Siberian Traps (*131*, *132*). (A) iceline extent, with proxy data (*42*, *43*). (B) pCO_2 levels with proxy data (*57*, *58*). (C) Equatorial average surface temperature from $\delta^{18}O$ (*51*). (D) global average surface temperature, against proxy curves of (*45*) (blue) and (*47*) (indigo).

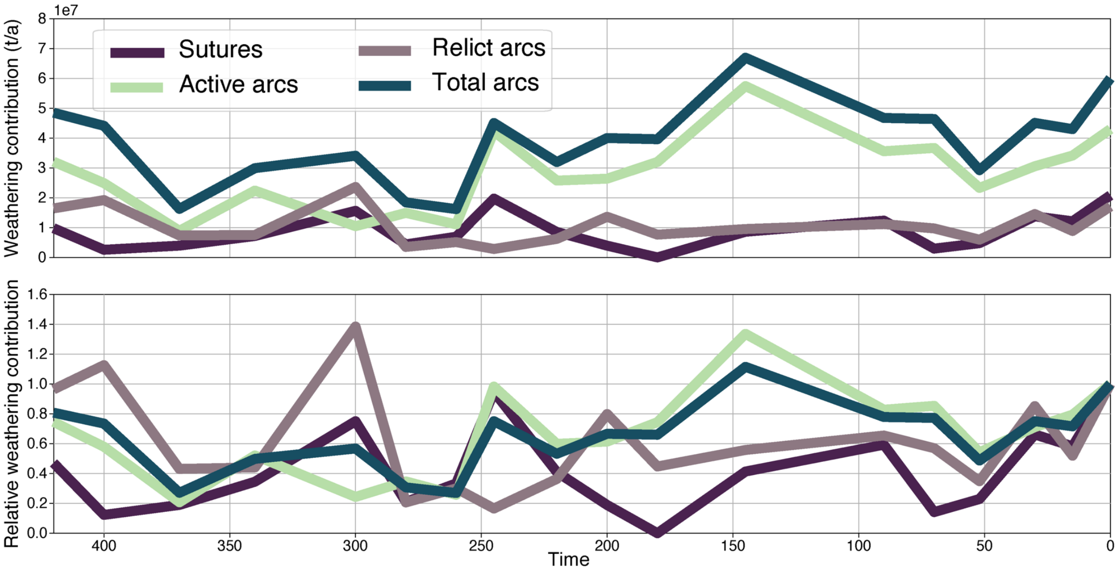

fig. S15: Contributions of our various geological features to the silicate weathering cycle. Values are taken from figs. S5–8.

Supplementary Table 1: Summary of weathering parameters used in our analysis (*40*, *88*). We used an implementation of a least-squares algorithm, comparing against present-day dissolved river loads, to find the optimised value within the given ranges.

Parameter	Purpose	Range of values	Optimized value
K	Inherent characteristic of mineral weathering and dependence on grain size	10^{-5}–10^{-2}	10^{-5}
K_w	Role of water flow	10^{-6}–10^{-3}	10^{-3}
σ	Effect of time on weathering rate	0.6–1.3	0.89
AEF	Weathering enhancement due to presence of peri-continental arcs	7–20	7
SEF	Weathering enhancement due to presence of ophiolite-bearing sutures	7–20	20

Supplementary Files

Data s1

Summary of degassing calculations and rates from different tectonic regions used to compile our degassing curve.

Data s2

pySCION model base-code. Requires python installation to run.
